# The Molecular Signature of the Stroma Response in Prostate Cancer-Induced Osteoblastic Bone Metastasis Highlights Expansion of Hematopoietic and Prostate Epithelial Stem Cell Niches

**DOI:** 10.1371/journal.pone.0114530

**Published:** 2014-12-08

**Authors:** Berna C. Özdemir, Janine Hensel, Chiara Secondini, Antoinette Wetterwald, Ruth Schwaninger, Achim Fleischmann, Wolfgang Raffelsberger, Olivier Poch, Mauro Delorenzi, Ramzi Temanni, Ian G. Mills, Gabri van der Pluijm, George N. Thalmann, Marco G. Cecchini

**Affiliations:** 1 Urology Research Laboratory, Department of Urology and Department of Clinical Research, University of Bern, Bern, Switzerland; 2 Institute of Pathology, University of Bern, Bern, Switzerland; 3 Institut Génétique Biologie Moléculaire Cellulaire (IGBMC), Strasbourg, France; 4 ICube UMR7357, University of Strasbourg, Strasbourg, France; 5 Ludwig Center for Cancer Research, Department of Oncology, University of Lausanne and Swiss Institute of Bioinformatics (SIB), Lausanne, Switzerland; 6 Biomedical Informatics Division, Sidra Medical and Research Center, Doha, Qatar; 7 Prostate Cancer Research Group, Norway Centre for Molecular Medicine (NCMM), University of Oslo, Oslo, Norway; 8 Department of Urology, Leiden University Medical Centre (LUMC), Leiden, The Netherlands; University of L′Aquila, Italy

## Abstract

The reciprocal interaction between cancer cells and the tissue-specific stroma is critical for primary and metastatic tumor growth progression. Prostate cancer cells colonize preferentially bone (osteotropism), where they alter the physiological balance between osteoblast-mediated bone formation and osteoclast-mediated bone resorption, and elicit prevalently an osteoblastic response (osteoinduction). The molecular cues provided by osteoblasts for the survival and growth of bone metastatic prostate cancer cells are largely unknown. We exploited the sufficient divergence between human and mouse RNA sequences together with redefinition of highly species-specific gene arrays by computer-aided and experimental exclusion of cross-hybridizing oligonucleotide probes. This strategy allowed the dissection of the stroma (mouse) from the cancer cell (human) transcriptome in bone metastasis xenograft models of human osteoinductive prostate cancer cells (VCaP and C4-2B). As a result, we generated the osteoblastic bone metastasis-associated stroma transcriptome (OB-BMST). Subtraction of genes shared by inflammation, wound healing and desmoplastic responses, and by the tissue type-independent stroma responses to a variety of non-osteotropic and osteotropic primary cancers generated a curated gene signature (“Core” OB-BMST) putatively representing the bone marrow/bone-specific stroma response to prostate cancer-induced, osteoblastic bone metastasis. The expression pattern of three representative Core OB-BMST genes (PTN, EPHA3 and FSCN1) seems to confirm the bone specificity of this response. A robust induction of genes involved in osteogenesis and angiogenesis dominates both the OB-BMST and Core OB-BMST. This translates in an amplification of hematopoietic and, remarkably, prostate epithelial stem cell niche components that may function as a self-reinforcing bone metastatic niche providing a growth support specific for osteoinductive prostate cancer cells. The induction of this combinatorial stem cell niche is a novel mechanism that may also explain cancer cell osteotropism and local interference with hematopoiesis (myelophthisis). Accordingly, these stem cell niche components may represent innovative therapeutic targets and/or serum biomarkers in osteoblastic bone metastasis.

## Introduction

Prostate cancer (PCa) is the most common solid cancer in men in the western world. Despite early detection and surgical treatment of the tumor, 10–20% of PCa patients show bone metastases at diagnosis [1] and >80% of advanced PCa patients have bone metastases at autopsy [2]. Bone metastases are the most important cause of morbidity in these patients and, once developed, are incurable.

In primary and metastatic cancers neoplastic cells closely interact with different cell types and the extracellular matrix (ECM) constituting the stroma compartment. This leads to activation of the stroma and, in turn, to the secretion of additional growth factors, matrix proteins and proteases, which further favor cancer cell proliferation and invasion. These heterogeneous and bi-directional interactions within the tumor tissue are fundamental for tumor growth progression [3]. Therefore, elucidation of the mechanism(s) determining the initiation and progression of metastatic growth is essential for the identification of novel therapeutic targets for prevention and/or treatment of cancer metastases.

In bone metastases activation of the bone marrow/bone (BM/B) stroma by cancer cells alters the physiological balance between osteoblast (OB)-mediated bone formation and osteoclast (OC)-mediated bone resorption, and interferes with hematopoiesis (myelophthisis). PCa elicits predominantly an OB response, with a consequent increase in bone formation (osteoinduction) and generation of osteosclerotic lesions [4]. Instead, mammary cancer (MCa) triggers preferentially an OC reaction, resulting in exaggerated bone resorption and generation of osteolytic lesions [5]. These opposite stromal reactions may underlie different growth support requirements between pro-osteolytic and osteoinductive cancer cells.

Factors released from the bone matrix during OC-mediated bone resorption fuel the proliferation of cancer cells, which stimulate further bone resorption, thereby leading to a self-perpetuating, positive feedback loop. This mechanism, known as the “Vicious Cycle” hypothesis of bone resorption and tumor growth in osteolytic bone metastases is a paradigmatic example of cancer cell-stroma interaction and has provided the rationale for interfering with the bone stroma support by inhibition of bone resorption [6]. However, the molecular cues provided by OBs for survival and growth of osteoinductive cancer cells have remained largely elusive [4].

Paget's “Seed & Soil” hypothesis [7] postulates that cancer cells (the “seed”) from the primary tumor can disseminate to various tissues, but succeed in establishing secondary growth only in those that are permissive for their survival and/or proliferative expansion (the “fertile soil”) [8]. Thus, the “Seed & Soil” hypothesis embodies the propensity of certain cancers to metastasize to specific tissues (tissue tropism). Paget was also the first to recognize that MCa metastasizes almost exclusively to the axial skeleton, site of the red marrow [7]. This observation suggests that active hematopoiesis may represent the fertile soil for seeding of cancer cells, which may therefore mimic hematopoietic stem cells (HSCs) for homing, survival and/or expansion in the BM/B [9]. This hypothesis, implying that the BM/B metastatic niche may match with the HSC niche, has been experimentally validated by showing that PCa cells hijack the HSC niche(s) for homing in the BM/B [10].

Most attempts to decipher the cancer gene signatures highlighting signaling pathways critical for cancer progression or predicting patient outcome have been performed by gene expression profiling in clinical samples of bulk tumor tissue. Obviously, this strategy cannot discriminate between cancer- and stroma-derived gene expression. Naef and Huelsken [11] have developed a method of “tissue compartment-specific transcriptional profiling” (TCTP), which allows simultaneous analysis of gene expression specific for the cancer cell and the stromal compartment *in situ*, without prior cell separation. This approach exploits differences between human and mouse RNA sequences and selects the most species-specific oligonucleotide probes by a computational mask. By a similar strategy we generated the osteoblastic bone metastasis-associated stroma transcriptome (OB-BMST) defining for the first time the global stroma response in bone xenograft models of osteoinductive PCa cells.

## Results

### Osteoinductive PCa cells alter the BM/B stroma transcriptome

**Figure 1:**
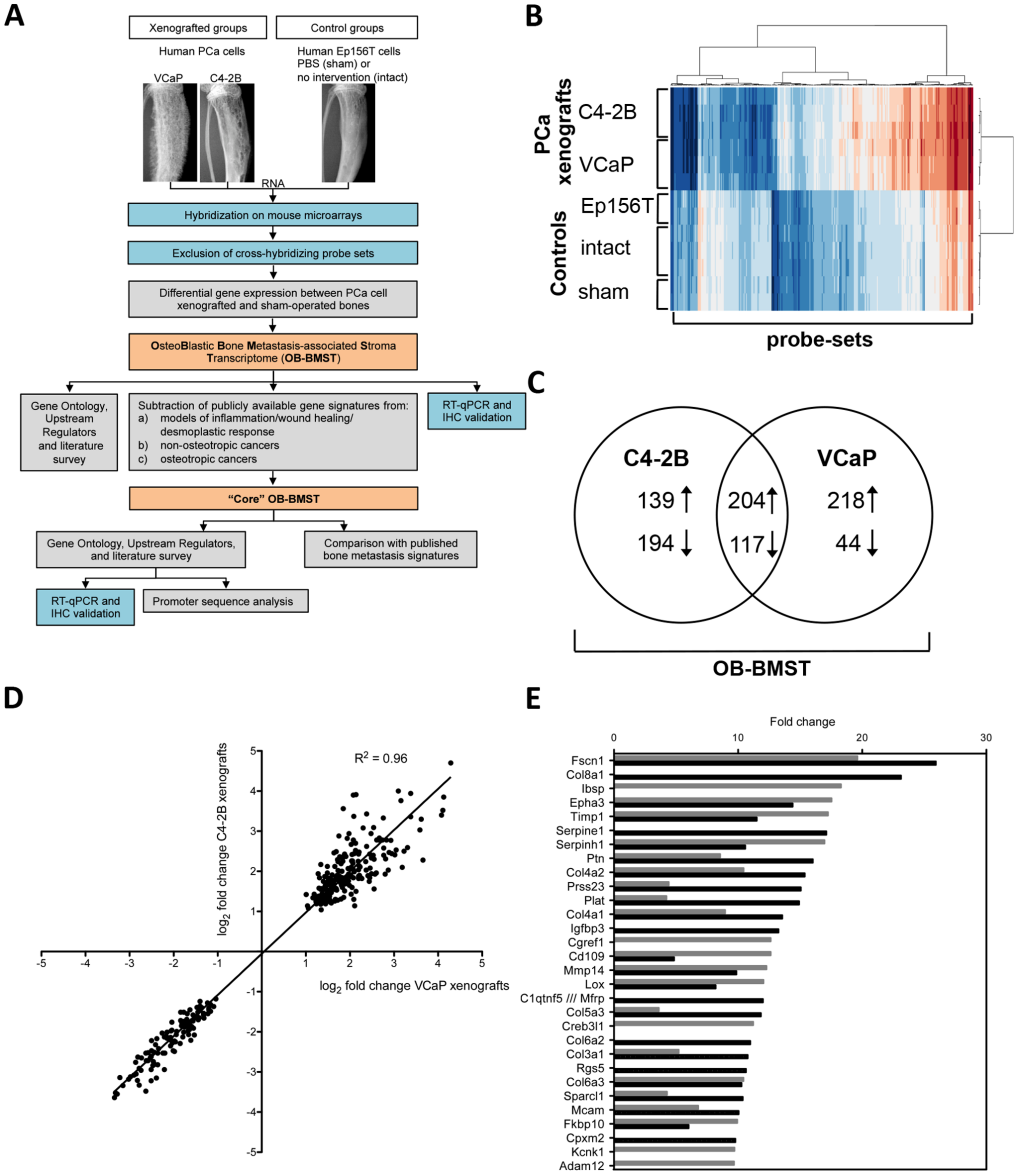
The gene expression pattern changes in bones xenografted with osteoinductive PCa cells. **A.** Flow chart outlining experimental (blue) and bioinformatics (grey) steps adopted to define a complete (OB-BMST) and a curated (“Core” OB-BMST) stroma response signature (orange). The first two experimental steps constitute the tissue compartment-specific transcriptional profiling (TCTP). **B.** Heatmap showing differentially expressed probe-sets between xenografted (C4-2B and VCaP) and control (Ep156T, intact and sham) bones. The expression level is color-coded: low expression is represented in blue, whereas high expression is represented in red. **C.** Venn diagram showing the number of overlapping and unique genes differentially expressed in C4-2B (FDR = 1E-05) and VCaP (FDR = 3E-05) xenografted bones. The sum of differentially expressed genes is referred to as the OB-BMST. **D.** Scatter plot showing log_2_ fold change of differentially expressed genes in C4-2B and VCaP xenografts. **E.** Top 30 up-regulated genes of the OB-BMST derived from C4-2B (black bars) and VCaP (grey bars) xenografted bones.

The strategy adopted to investigate transcriptome changes that occur specifically in the stroma compartment of bones xenografted with human osteoinductive prostate cancer cells is outlined in **Fig. 1A**.

The differentially expressed probe sets clearly separate cancer-cell xenografted bones from control bones, as highlighted by hierarchical clustering (**Fig. 1B**). The heatmap shows homogeneous clustering within the xenografts, but the dendrogram still indicates that the C4-2B and VCaP xenografts separate in two branches. All control samples also cluster together, suggesting that surgical intervention and inoculation of non-tumorigenic, normal human prostate epithelial cells (Ep156T) did not cause major modifications in gene expression. Additionally, the heatmap indicates that in xenografts the prevalent part of genes (64%) were up-regulated.

When compared to the sham-operated bones, 654 genes are differentially expressed in C4-2B xenografts (false discovery rate, FDR≤1E-05) and 583 genes in VCaP xenografts (FDR≤3E-05) (**Fig. 1C**, **S1 Table**). The sum of these differentially expressed genes will be referred to as the OB-BMST.

A total of 321 genes from the OB-BMST are common to both C4-2B and VCaP xenografts (**Fig. 1C**, **S1 Table**). The scatter plot of log_2_ fold change for both xenografts shows a significant correlation (R^2^ = 0.96) (**Fig. 1D**), indicating a complete concordance of gene regulation for both models of osteoblastic bone metastasis. However, within the OB-BMST, 333 and 262 genes are unique for C4-2B and VCaP, respectively. Differences in osteoinductive potential (VCaP>C4-2B) and/or in growth kinetics (C4-2B>VCaP) may explain this partial diversity of stroma reaction induced by the two cell lines. Nevertheless, among the top 30 most up-regulated genes 18 are shared by C4-2B and VCaP xenografts (**Fig. 1E**, **S1 Table**), indicating that both cell lines are comparable in inducing the predominant stroma reaction.

### The OB-BMST associates with myoepithelial/myofibroblast signature

To identify the cell type primarily responsible for the OB-BMST expression, we compared the up-regulated genes of the OB-BMST with gene signatures previously derived from specific stroma cell populations in mammary tumors [12]. This analysis shows that, the OB-BMST mainly overlaps with the myoepithelial/myofibroblast signature and, to a minor extent, with the fibroblast and EC signatures (**S1 Figure**).

### A fraction of the OB-BMST is not specific for the BM/B response to osteoinductive PCa cells

The analysis of the expression of a 7-gene set of highly up-regulated OB-BMST genes, namely periostin (*Postn*), asporin (*Aspn*), SPARC-like 1 (*Sparcl1*), melanoma cell adhesion molecule (*Mcam*), platelet derived growth factor receptor beta (*Pdgfrb*), fascin homolog 1 (*Fscn1*) and prostate transmembrane protein, androgen induced 1 (*Pmepa1*) revealed that these genes are not only induced in the stroma of bones xenografted with C4-2B and VCaP cells (**S2A Figure**), but also in orthotopic VCaP xenografts (**S2B Figure**) and in ectopic (**S2C Figure**) xenografts of both C4-2B and VCaP cells. In addition, the expression of the 7-gene set is induced also in bones xenografted with the pro-osteolytic PCa cell line PC-3 (**S2D Figure**). Furthermore, ASPN and POSTN expression is not only increased in the stroma of bone metastatic human PCa, but also of primary PCa and of bone metastatic human MCa (**S3 Figure**).

Taken together, the results above suggest that a fraction of the OB-BMST is not specific for the BM/B response to osteoinductive PCa cells. However, the finding that proteins encoded by genes of the OB-BMST are overexpressed also in the stroma of human primary and bone metastatic tumors underscores the translational value of the OB-BMST.

### A fraction of the OB-BMST is specific for the BM/B response to osteoinductive PCa cells

To identify the OB-BMST component specific to the osteoblastic bone metastases, we subtracted from the OB-BMST stroma gene signatures derived from inflammatory/wound healing and desmoplastic responses, and from non-osteotropic and osteotropic cancers (**Fig. 1A**).

This strategy, referred to, thereafter, as “curation”, has led to the identification of 4 major components within the OB-BMST: *1)* a component putatively specific for the BM/B stroma response to osteoinductive PCa cells, from now on referred to as “Core OB-BMST”, *2)* a component shared with “inflammatory/wound healing/desmoplastic” response signatures, *3)* a component possibly representing a “universal” response to cancer cells and *4)* a candidate “osteotropic” signature.

**Figure 2:**
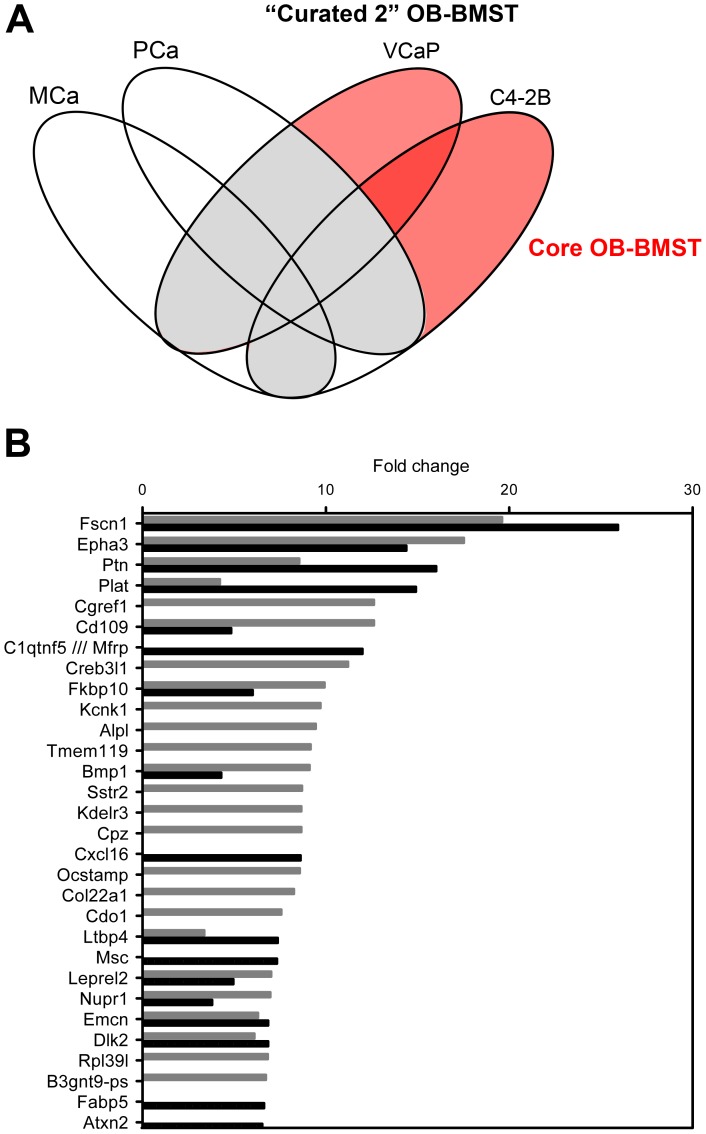
The Core OB-BMST represents the fraction putatively specific for the BM/B response to osteoinductive PCa cells. **A.** Four-set Venn diagram showing the comparison of primary MCa, PCa with the OB-BMST after subtraction of gene signatures derived from desmoplastic, wound-healing, inflammatory and non-osteotropic cancers ( =  “Curated 2” OB-BMST, sum of grey and red areas). The red shaded area is referred to as “Core OB-BMST” (complete list reported in S2 Table), genes of the grey area represent a potential osteotropic signature (complete list reported in S6 Table). **B.** Top 30 up-regulated genes of the Core OB-BMST derived from C4-2B (black bars) and VCaP (grey bars) xenografted bones.

The Core OB-BMST, covering 72.6% of the OB-BMST, consists of 336 up-regulated and 298 down-regulated genes. Of these, 109 and 93, respectively, are common to both C4-2B and VCaP xenografts (**Fig. 2A**, **S2 Table**). These genes are likely to be restricted to the BM/B stroma reaction to osteoinductive cancer cell growth. However, the specificity of the Core OB-BMST should be considered with caution since our subtractive strategy was limited to publicly available gene signatures and did not consider studies concerning single gene and/or protein expression in a variety of cancers.

The top 10 genes of the Core OB-BMST list (**Fig. 2B**, **S2 Table**) are conserved from the top 30 most up-regulated genes of the OB-BMST (**Fig. 1E**, **S1 Table**). Among the top 30 genes of the Core OB-BMST, 12 are common to C4-2B and VCaP xenografts, while 13 and 5 are unique of VCaP and C4-2B, respectively. Thus, in contrast to the OB-BMST top 30-gene list, the Core OB-BMST is primarily induced by VCaP cells. Most likely, this is the consequence of the more robust OB response induced by these cells, as compared to C4-2B (**Fig. 1A**).

### Angiogenesis and osteogenesis are the key processes represented in the OB-BMST and Core OB-BMST

**Figure 3:**
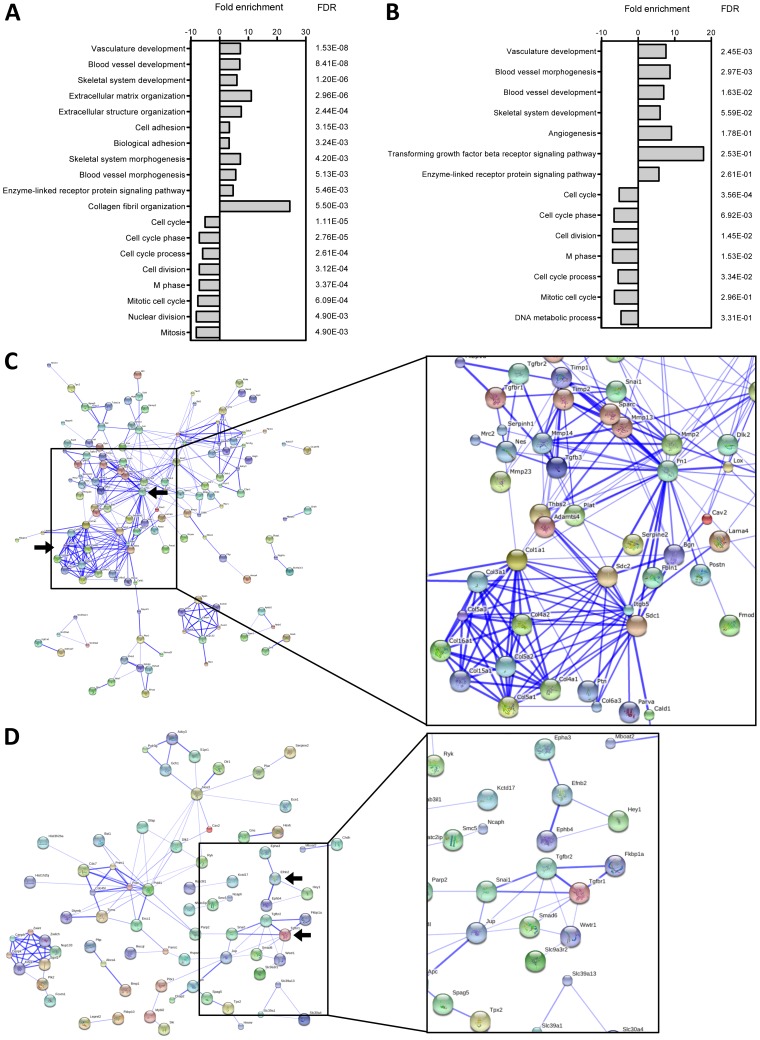
Enriched GO terms and protein interaction networks within the “common” OBMST and Core OB-BMST. GO terms enriched in the “common” OB-BMST (FDR≤5.50E-03) (**A**) and in the “common” Core OB-BMST (FDR≤5.0E-01) (**B**). Protein interaction networks by STRING analysis in the “common” OB-BMST (**C**) and in the “common” Core OB-BMST (**D**). The thickness of lines correlates positively with the confidence score that was obtained for each protein interaction. Abbreviation: FDR, false discovery rate.

GO terms analysis shows that up-regulated genes in both the OB-BMST and Core OB-BMST common to VCaP and C4-2B (“common” OB-BMST/Core OB-BMST) are highly associated (FDR<0.05) with annotations terms related to angiogenesis, skeletal system development and enzyme-linked receptor protein signaling pathway (**Fig. 3 A and B**). ECM organization and cell adhesion are also highly represented in the OB-BMST, while TGFβ receptor signaling appears as additional term in the Core OB-BMST. Down-regulated genes are mainly grouped in GO terms related to cell cycle (**Fig. 3A and B**). Most likely, the complete lack of hematopoiesis in the BM spaces invaded by cancer cells (myelophthisis) is responsible for this phenomenon.

The protein network analysis reveals fibronectin 1 (Fn1) as the central node of the OB-BMST, with 31 interaction partners involved in ECM remodeling (e.g. Bgn, Fbln1, Fmod, Adamts4, Timp1, Postn, Mmp14), skeletal system development (Sparc, Col1a1, Mmp13, Serpinh1), Wnt signaling (Sdc1, Ryk, Jup), cell adhesion (Mcam, Thbs2, Itgb5), angiogenesis (Jun, Nos3), wound healing (Gfap, Lox) and epithelial-to-mesenchymal transition (Tgfβ3, Snai1). A second prominent network consists of 10 collagen family members and is linked to Fn1 by Sdc1 and 2 (**Fig. 3C**). Analysis of protein interaction networks in the Core OB-BMST points at Tgfβ receptor and Ephrin signaling (**Fig. 3D**).

Among the 67 activated upstream regulators identified for the “common” OB-BMST, 15 represent growth factors (Tgfβ1, Ctgf, Fgf2, Agt, Igf, Gdf2, Vegfa, Bmp2, Tgfβ3, Bmp4, Igf1, Jag1, Lep, Pdgfb, Inhba) and 6 represent cytokines (Prl, Idn1, Il17a, Csf1, Osm, Wnt1) (**S3 Table**). For the “common” Core OB-BMST, the number of activated biological upstream regulators is considerably restricted (Tgfβ1, Ephb4, Kdm5b, Nupr1, Igfr1 and Fgf2). Only Tgfβ1 and Fgf2 are retained from the OB-BMST, and Ephb4 emerges as an activator of the canonical Tgfβ pathway (**Table 1**). Tgfβ1 has the highest activation score, stringency and largest number of predicted target molecules for both transcriptomes (**Table 1** **and** **S3 Table**).

**Table 1:** Activated upstream regulators of the “common” Core OB-BMST.

Gene Symbol	Gene Name	Fold Change	Molecule Type	Activation z-score	*p*-value of overlap	Target molecules in dataset
Tgfβ1	transforming growth factor, beta 1		growth factor	3.47	3.54E-12	Abca1,Anpep,Arf4,Bmp1, Cav2,Cdc,Cnn3,Col16a1, Ece1,Elk3,Fermt2, Fscn1,Gfap,Gns,Hes1,Hexb,Hey1,Jup,Ltbp3,Mboat2, Mphosph9,Mybl2,Myof,Nab2,Nos3,Nupr1,Olr1,Pdlim4, Plat,Plk2,Plod1,Plscr1,Pold1,Prim1,Rad51ap1,Ramp2, Serpine2,Ski,Slc39a1, Smad6,Snai1,Tgfβr1,Tgfβr2,Tyms,Zwint
Ephb4	EPH receptor B4	3.1	kinase	2.00	9.51E-04	Smad6,Tek,Tgfβr1,Tgfβr2
Kdm5b	lysine (K)-specific demethylase 5B		transcription regulator	2.00	2.03E-02	Hmmr,Ncaph,Recql,Smc5
Nupr1	nuclear protein, transcriptional regulator, 1	6.97	transcription regulator	2.12	3.44E-02	B3gnt5,Brcc3,Gch1,Gk, Gtse1,Mms22l,Nupr1,Spag5,Stil
Igf1r	insulin-like growth factor 1 receptor		transmembrane receptor	2.00	3.70E-02	Nos3,Plat,Prkcdbp,Snai1, Tyms
Fgf2	fibroblast growth factor 2 (basic)		growth factor	2.35	8.39E-02	Anpep,Efnb2,Gfap,Nos3, Plat,Snai1

The analysis of over-represented sequence motifs in the promoters of the genes of the Core OB-BMST shows that only the up-regulated genes have significant motif over-representation, namely for Foxo4, Meis1 and Maz (**Table 2**). The processes associated with the Foxo4-, Meis1- and Maz-linked genes are angiogenesis and vasculogenesis (*P* = 10E-09), cell junction assembly and organization (*P* = 10E-05) and collagen fibril organization and extracellular matrix organization (*P* = 10E-03).

**Table 2:** Promoter sequence motifs overexpressed in the common Core OB-BMST.

Transcription factor	Genes	Enrichment factor	*p*-value	Biological processes
V$MAZ_Q6	NAB2, ELK3, HES1, S100A16, PRKCDBP, RAMP2, STC1, BMP1, ITM2C, MRC2, FKBP10, ABCA1, ARF4, JUP, SLCO2A1, SLC39A13, PLAT, CNN3, PHLDB1, CD109, ANXA6	1.4	1.36E-06	Collagen fibril organization and extracellular matrix organization (10E-03)
V$MEIS1_01	SLC30A4, ELK3, COPZ2, RAMP2, BMP1, PTN, PDLIM4, EPHA3, JUP, HOXA3, PHLDB1, SNAI1	1.3	3.20E-05	Cell junction assembly and organization (10E-05)
V$FOXO4_01	COLEC12, TEK, APC, NOS3, STC1, GNS, PTN, PDLIM4, MRC2, SMAD6, PITX1, EMCN, JUP, HOXA3, HEY1, CAV2, RAMP3	1.3	5.61E-05	Angiogenesis and vasculogenesis (10E-09 and 10E-10)

Collectively, the findings above strongly suggest angiogenesis and osteogenesis as major biological processes and Tgfβ as the major signaling pathway involved in osteoblastic bone metastasis.

### The OB-BMST and Core OB-BMST partially overlap with “stem cell niche” signatures

The literature survey for 96 of the most up-regulated genes of the Core OB-BMST shows that 32 genes (33.3%) are involved in OB recruitment/function, 6 genes (6.3%) in OC recruitment/function, 34 genes (35.4%) in EC recruitment/function and 13 genes (13.5%) in MSC differentiation/function. Notably, 10 genes (10.4%) are documented HSC niche components and 9 genes (9.4%) cancer cell niche components. It has to be considered that single genes could be assigned to more than one category. Forty-one genes (42.7%) are unrelated to any of the categories above (**Table 3**, **S4 Table**).

**Table 3:** Literature survey of up-regulated Core OB-BMST genes.

Gene symbol	Gene name	Fold change VCaP/C4-2B	FDR VCaP/C4-2B	Functions	References
**Hematopoietic stem cell niche components**					
*Abca1*	ATP-binding cassette, sub-family A (ABC1), member 1	3.32/4.05	6.73E-06/4.65E-06	Regulation of HSC niche	Westerterp *et al.*, 2012
*Cdh2*	Cadherin 2	5.94/1.86	1.68E-05/1.81E-02	Mediates homophilic adhesion to osteoblasts in the HSC niche	Zhang *et al*., 2003; Arai *et al*., 2102
*Epha3*	Eph receptor A3	17.53/14.39	1.20E-06/1.51E-06	Homing factor for stem cells to the BM	Ting *et al.*, 2010
*Lamb1*	Laminin B1 subunit 1	4.38/5.72	2.21E-05/3.93E-06	Laminins facilitate survival and self-renewal of pluripotent stem cells	Gu *et al*, 2003; Rodin *et al.*, 2010
*Nos3*	Nitric oxide synthase 3, (endothelial cell)	3.12/3.22	6.73E-06/2.77E-06	Regulates maintenance and mobilization of stem cells in the BM	Aicher *et al.*, 2003; North *et al*., 2009
*Olfml3*	Olfactomedin-like 3	5.44/3.37	1.20E-06/5.90E-06	Regulates assembly of HSC perivascular niche	Miljkovic-Licina *et al.*, 2012
*Ptn*	Pleiotrophin	8.55/16.02	1.20E-06/3.08E-07	Regulates the maintenance of the HSC pool; homing factor for stem cells to the BM	Himburg *et al.*, 2012
*S1pr1*	Sphingosine-1 phosphate receptor 1	4.05/3.77	1.96E-06/1.75E-06	Expressed on HSCs; S1P ( = ligand) facilitates the egress of committed hematopoietic progenitors from the BM into the blood	Juarez *et al.*, 2012
*Sstr2*	Somatostatin receptor 2	8.70/1.98	1.59E-07/1.45E-04	Expressed on HSCs, involved in BM homing	Oomen *et al.*, 2002
*Tek*	Endothelial-specific receptor tyrosine kinase	3.48/4.57	6.73E-06/1.10E-06	Expressed in HSCs; maintains quiescent status of HSCs	Yano *et al.*, 1997; Martin *et al.*, 2008
**Cancer cell niche components**					
*Bmp1*	Bone morphogenetic protein 1	9.11/4.29	1.34E-06/3.84E-06	Promotes proteolytic activation of lysyl oxidase	Maruhashi *et al.*, 2010; Erler *et al*., 2009
*Epha3*	Eph receptor A3	17.53/14.39	1.20E-06/1.51E-06	Promotes angiogenesis, expressed on tumor-initiating cell population, maintains tumor cells in a less differentiated state	Xi *et al.*, 2012; Day *et al.*, 2013
*Ephb4*	Eph receptor B4	3.10/3.37	1.17E-05/3.84E-06	Deregulated Ephb4-ephrinb2 signaling may contribute to the acquisition of a metastatic phenotype; modulates angio-/lymph-angiogenesis	Kaenel *et al.*, 2011; Abéngozar *et al.*, 2012
*Lamb1*	Laminin B1, subunit 1	4.38/5.72	2.21E-05/3.93E-06	Displays anti-adhesive functions and has potential implications for cell migration during matrix remodeling; angiogenesis	Santos-Valle *et al.*, 2012; Patarroyo *et al.*, 2002; Ghajar *et al*., 2013
*Ltbp4*	Latent transforming growth factor beta binding protein 4	3.37/7.37	6.73E-06/3.08E-07	Modulates activation of latent TGFβ	Ghajar *et al.,* 2013
*Nid1*	Nidogen 1	-/5.98	-/2.74E-06	Overexpressed in BM-like microvascular niche *in vitro*	Ghajar *et al.,* 2013
*Olfml3*	Olfactomedin-like 3	5.44/3.37	1.20E-06/5.90E-06	Promotes angiogenesis and tumor growth	Miljkovic-Licina *et al.*, 2012
*Ptn*	Pleiotrophin	8.55/16.02	1.20E-06/3.08E-07	Stimulates angiogenesis; activates CAFs; stimulates cancer cell growth	Perez-Pinera *et al.*, 2007; Diamantopoulou *et al.*, 2012
*S1pr1*	Sphingosine-1 phosphate receptor 1	4.05/3.77	1.96E-06/1.75E-06	Regulates pre-metastatic niche; angiogenesis	Deng *et al.*, 2012; Yang *et al.*, 2013

**Note**: 96 genes corresponding to all genes more than 3 fold induced in both xenografts and the top 30 of VCaP and C4-2B xenografts were reviewed. Complete references can be found in S4 Table.

Abbreviations: BM, bone marrow; CAF, cancer-associated fibroblast; FDR, false discovery rate; HSC, hematopoietic stem cell.

**Figure 4:**
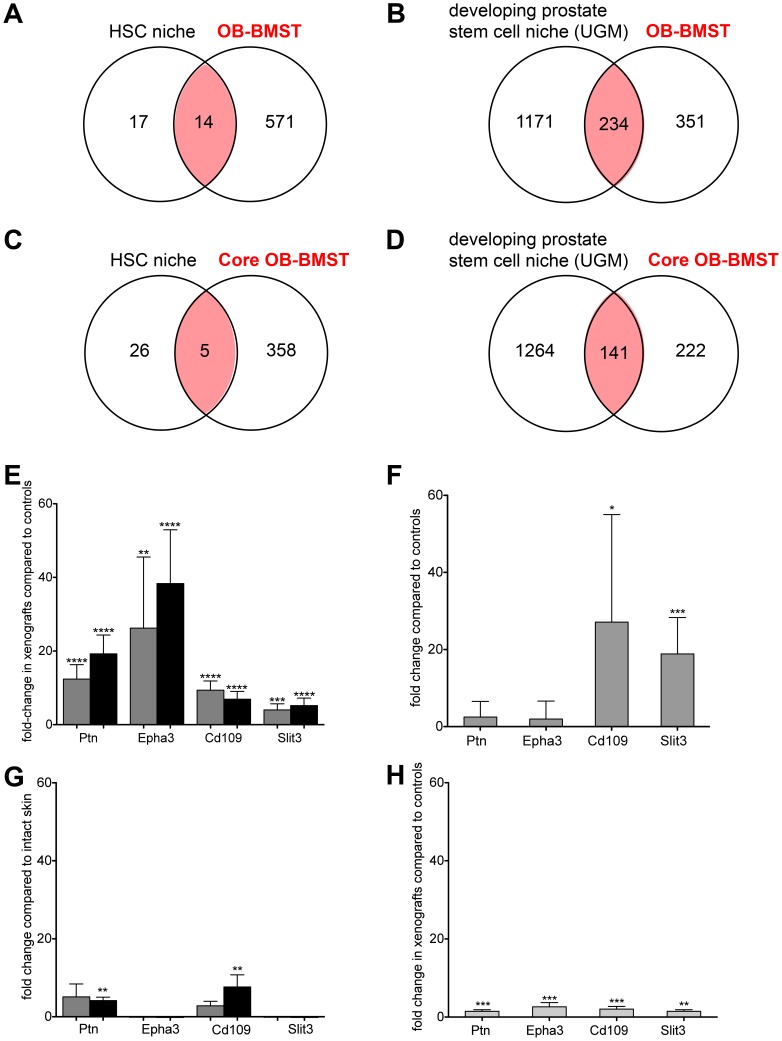
The OB-BMST and Core OB-BMST contain genes from stem cell niche signatures. Venn diagrams showing the number of overlapping OB-BMST (**A** and **B**) and Core OB-BMST (**C** and **D**) genes with gene signatures derived from the hematopoietic (**A** and **C**) and the developing prostate (**B** and **D**) stem cell niches. Relative expression levels of *Ptn*, *Epha3*, *Cd109* and *Slit3*. **E.** VCaP (grey, *n* = 3) and C4-2B (black, *n* = 4) intra-osseous xenografts; values are shown as fold change (mean ± SD) relative to contralateral and sham-operated bones. **F.** VCaP (grey, *n* = 5) orthotopic xenografts; values are shown as fold change (mean ± SD) relative to intact and sham-operated prostate. **G.** VCaP (grey, *n* = 3) and C4-2B (black, *n* = 5) subcutaneous xenografts; values are shown as fold change (mean ± SD) relative to intact skin. **H.** PC-3 (light grey, *n* = 6) intra-osseous xenografts; values are shown as fold change (mean ± SD) relative to contralateral and sham-operated bones (*n* = 3–4). *, *P*<0.01; **, *P*<0.001; ***, *P*<0.0001; ****, *P*<0.0001. Abbreviations: HSCs, hematopoietic stem cells; UGM urogenital mesenchyme; *Ptn*, pleiotrophin; *Epha3*, Eph receptor a3; *Slit3*, slit homolog 3.

To further corroborate the contribution of SC niche-related genes to the osteoblastic response, we compared both the OB-BMST and the Core OB-BMST with two SC niche signatures publicly available for the HSC [13] and for the developing prostate (uro-genital mesenchyme, UGM) [14]. The OB-BMST contains 14 genes (45%) of the 31 HSC-gene signature [13] and 208 genes (14.8%) of the 1405 UGM-gene signature (**Fig. 4A and B**). As an effect of the curation, 5 (16%) of the HSC genes and 141 (11%) of UGM genes are retained in the Core OB-BMST (**Fig.4C and D**, **Table 4** and **S5 Table**). Importantly, 37% and 42% of the up-regulated genes of OB-BMST and Core OB-BMST, respectively, represent genes up-regulated also in the UGM signature.

**Table 4:** Core OB-BMST genes overlapping with the hematopoietic and developing prostate stem cell niche signatures.

Hematopoietic stem cell niche										
*Hes1*	*Ptn*	*Slc8a1*	*Slco2a1*	*Tspan6*						
**Developing prostate stem cell niche**										
*Antxr2*	*Anxa5*	*Anxa6*	*Apbb1*	*Aplnr*	*Aplp1*	*Arhgef25*	*B3gnt9-ps*	*B4galt2*	*Bicc1*	*Casp12*
*Ccdc80*	*Cd200*	*Cd40*	*Cd93*	*Cdh15*	*Cdh2*	*Cdo1*	*Cdr2l*	*Cgref1*	*Chst2*	*Cnn3*
*Col16a1*	*Copz2*	*Cplx1*	*Creb3l1*	*Cspg4*	*Cxx1a*	*Cxx1b*	*Cygb*	*Cyp7b1*	*Ddr2*	*Dnm1*
*Ednrb*	*Egfl7*	*Eln*	*Emcn*	*Eng*	*Enpep*	*Epb4.1l2*	*Epha3*	*Fabp7*	*Fam181b*	*Fcgrt*
*Fermt2*	*Fgfr1*	*Fibin*	*Fkbp10*	*Fkbp7*	*Fkbp9*	*Foxc2*	*Gas1*	*Gdf10*	*Gimap6*	*Gja4*
*Gli1*	*Gli2*	*Hey1*	*Hip1*	*Il6st*	*Itm2a*	*Jam2*	*Kdelr3*	*Ldb2*	*Lepre1*	*Leprel2*
*Lifr*	*LOC100862618*	*Lpar1*	*Mageh1*	*Matn2*	*Mrc2*	*Msc*	*Ndrg4*	*Nid1*	*Nos3*	*Npdc1*
*Ntn1*	*Olfml3*	*Pcdhga1*	*Pcdhga2*	*Pcdhga3*	*Pcdhga4*	*Pcdhga5*	*Pcdhga6*	*Pcdhga7*	*Pcdhga8*	*Pcdhga9*
*Pcdhga10*	*Pcdhga11*	*Pcdhga12*	*Pcdhgb1*	*Pcdhgb2*	*Pcdhgb4*	*Pcdhgb5*	*Pcdhgb6*	*Pcdhgb7*	*Pcdhgb8*	*Pcdhgc3*
*Pcdhgc4*	*Pcdhgc5*	*Phldb1*	*Phldb2*	*Pitx1*	*Pkd2*	*Plat*	*Plod1*	*Plvap*	*Pou3f1*	*Prkcdbp*
*Ptn*	*Ptprz1*	*Rab13*	*Ramp2*	*Rapgef4*	*Rerg*	*Rftn2*	*Rhoj*	*Sdc3*	*Sec16b*	*Selm*
*Slc22a17*	*Slc2a10*	*Slc8a1*	*Slc9a3r2*	*Slit3*	*Snai1*	*Sox18*	*Spred1*	*Sspn*	*St6galnac4*	*Stmn3*
*Tbx2*	*Tek*	*Tgfbr2*	*Tmem119*	*Tmem45a*	*Tspan4*	*Ttyh2*	*Vasn*	*Wwtr1*		

**Note**: Genes in bold are present in both stem cell niche signatures.

### The Core OB-BMST SC niche gene *Epha3* is specific for the BM/B stroma reaction in osteoblastic bone metastasis

The up-regulation of 4 representative SC niche genes, namely pleiotrophin (*Ptn*), Eph receptor a3 (*Epha3*), *Cd109* and Slit homolog 3 (*Slit3*) in VCaP and C4-2B bone xenografts is confirmed by RT-qPCR analysis (**Fig. 4E**). *Slit3* and, to a minor extent, *Cd109*, are significantly up-regulated also in the stroma of orthothopic VCaP (**Fig. 4F**). *Ptn* and, *Cd109* are significantly up-regulated also in the stroma of subcutaneous C4-2B xenografts (**Fig. 4G**). In contrast, none of the 4 SC niche genes are significantly induced in the stroma of subcutaneous VCaP xenografts (**Fig. 4G**). A significant induction of these genes is also shown in the stroma of bone xenografts of the pro-osteolytic PC-3 cells (**Fig. 4H**), yet this is negligible when compared to the induction in the stroma of bones xenografted with osteoinductive C4-2B and VCaP cells. These results indicate that, at least in cancer cell xenografts, induction of *Epha3* expression is the most specific for the osteoblastic response.

### Expression of the Core OB-BMST proteins PTN, EPHA3 and FSCN1 is restricted to human PCa bone metastasis

In order to verify the translational value of Core OB-BMST genes, we investigated the protein expression of the two SC-niche components PTN and EPHA3, and of the most up-regulated gene FSCN1 in normal prostate and bone, and in primary and bone metastatic PCa.

**Figure 5:**
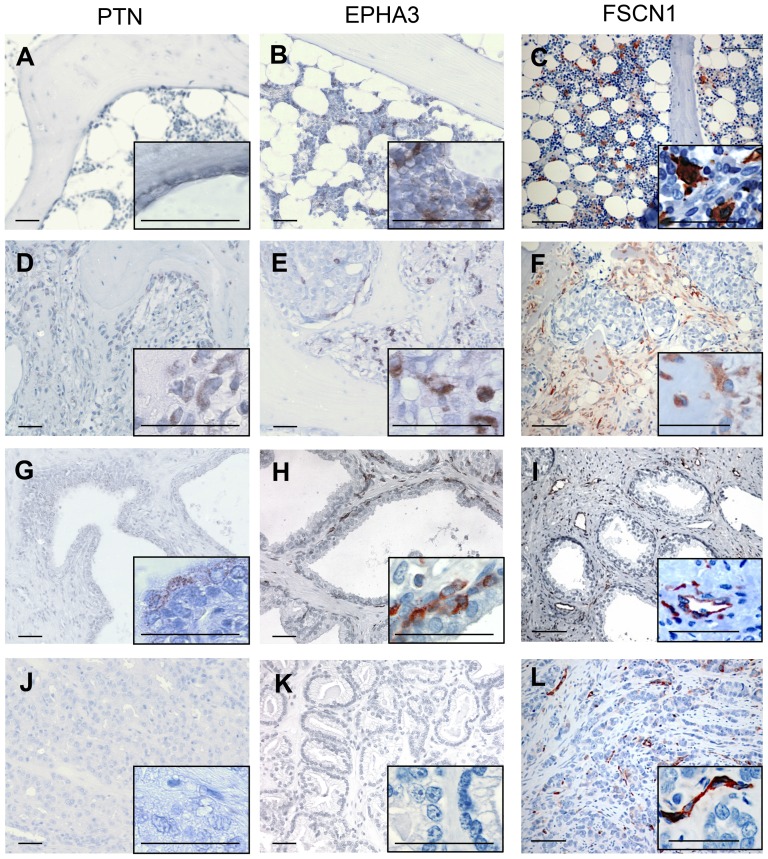
PTN, EPHA3 and FSCN1 protein expression is induced in human bone metastatic, but not primary PCa. Immunohistochemical detection of PTN (**A**, **D**, **G** and **J**), EPHA3 (**B**, **E**, **H** and **K**) and FSCN1 (**C**, **F**, **I** and **L**) in normal bone (**A**, **B** and **C**), in osteoblastic PCa bone metastasis (**D**, **E** and **F**), in normal prostate (**G**, **H** and **I**) and in primary PCa (**J**, **K** and **L**). Insets represent a higher magnification of selected areas. Scale bar = 50* µ*m. Abbreviations: PTN, pleiotrophin; EPHA3, Eph receptor A3; FSCN1, fascin homolog 1.

Normal bone and hematopoietic marrow (**Fig. 5A**) are devoid of PTN immunoreactivity. In bone metastases (**Fig. 5D**), OBs and newly embedded osteocytes are PTN-positive, but only in areas of cancer cell infiltration. Cancer cells are mostly negative, with the exception of few cells in proximity of bone forming OBs. In normal prostate (**Fig. 5G**) isolated clusters of luminal cells of some acini are PTN-positive, whereas the majority of acini are devoid of PTN immunoreactivity. Sections of PCa (**Fig. 5J**) are mostly negative, with the exception of few cancer cells in areas of high Gleason grade (not shown).

Strongly EPHA3-immunoreactive stellate-like cells are found scattered within normal BM (**Fig. 5B**) and, with increased density, in the tumor stroma of bone metastases (**Fig. 5E**). OBs, osteocytes and OCs are negative, as well as the majority of cancer cells. In normal prostate approximately half of the acini are EPHA3 negative, whereas in the other half discrete portions of the basal layer are positive (**Fig. 5H**). Positive staining is also found in rare myofibroblasts. In contrast, in primary PCa, cancer cells of all neoplastic acini are invariably EPHA3 negative, with no modification of the staining pattern in the stroma compartment (**Fig. 5K**).

In normal bone stellate cells within the hematopoietic marrow are strongly FSCN1-positive (**Fig. 5C**), but OBs, lining cells, osteocytes and OCs are FSCN1-negative. In contrast, in PCa bone metastasis (**Fig. 5F**), active OBs, osteocytes and fibroblast-like stromal cells surrounding areas of cancer cell growth are FSCN1-positive, while cancer cells are negative. In normal prostate, (**Fig. 5I**) FSCN1 immunoreactivity is detected in ECs of small vessels and in scattered, fibroblast-like cells. In PCa (**Fig. 5L**) the expression pattern of FSCN1 is similar to normal prostate tissue.

These findings further support the translational value of the OB-BMST and demonstrate that the Core OB-BMST contains genes that are specifically induced in the stroma of bone metastatic, but not of primary PCa.

### The OB-BMST also contains gene signatures not unique to osteoblastic bone metastasis

As outlined above, the curation strategy has led to the definition of 3 additional components within the OB-BMST.

One component, covering 8.7% of the OB-BMST genes, matches with previously generated, inflammatory, wound healing and desmoplastic response signatures [15]–[19] (**S6 Table**).

Another component covers 8.8% of the OB-BMST and shares genes with signatures previously retrieved from non-osteotropic cancers such as gastric [20], pancreatic [21], [22], colorectal [23] and esophageal [24], [25] (**S6 Table**).

The last component covers 9.9% of the OB-BMST and shares 88 genes with signatures obtained from primary MCa [12], [26]–[29] and PCa [29]–[33], known for their high propensity to metastasize to bone (osteotropism) [4], [5] (**Fig. 2A** **and** **S6 Table**). This component may represent a signature predicting disease progression as bone metastasis. Unfortunately, this possibility could not be validated in PCa since no gene expression datasets for metastatic outcome of primary PCa are publically available.

## Discussion

This is the first comprehensive transcriptome analysis defining the BM/B stroma reaction in xenograft models PCa-induced osteoblastic bone metastasis. The stroma specificity of this transcriptome, designated as OB-BMST, has been provided by the TCTP [11], a method allowing the analytical dissection of tumor stroma and cancer cell transcriptomes *in situ*, without physical separation of the two compartments.

### Osteogenesis and angiogenesis are the key processes in the OB-BMST and Core OB-BMST

The GO terms, the principal upstream regulators and the major effector cells of the OB-BMST and Core OB-BMST strongly indicate that osteogenesis and angiogenesis are the predominant processes in the BM/B stroma reaction of PCa-induced, osteoblastic bone metastasis. The scarcity of genes related to OCs (4 genes encoding inhibitors of OC recruitment/activity and only two genes promoting OC recruitment) in the Core OB-BMST and the lack of genes encoding master factors stimulating OC recruitment (Rankl, Csf-1 and IL-8) in the OB-BMST further support the notion that, in osteoblastic bone metastases, there is no increase in OC recruitment [34].

#### Osteogenesis

The contribution of osteogenesis to the OB-BMST/Core OB-BMST could be anticipated and confirms the robustness of the two mouse models of osteoblastic bone metastasis adopted in this study.

Markers and/or effectors of MSC and OB recruitment and function are highly enriched in the Core OB-BMST, where they represent 40% of the 96 most up-regulated genes. This indicates that the Core OB-BMST better illustrates the bone context-specific, stromal response to osteoinductive cancer cells than the original OB-BMST. Furthermore, the finding that the osteogenesis-related genes among the top 30 Core OB-BMST genes are predominantly induced by VCaP cells seems to underscore the higher osteoinductive potential of these cells, as compared to C4-2B.

Cancer-associated fibroblasts (CAFs) are an additional cell population contributing to the OB-BMST as indicated by: *a)* the association with a previously retrieved myofibroblasts/fibroblasts gene signature [12], *b)* the enrichment of CAF markers (i.e., Pdgfrb and Sparc) and recruiting factors (Tgfβ1, Tgfβ3, Fgf2 and Pdgfbb) [35], *c)* the pivotal position of CAF-derived ECM proteins (Fn1 and collagens) [36] in the protein network and *d)* the expression of ASPN and POSTN by myofibroblasts in primary and bone metastatic PCa. This finding is in agreement with a previous study reporting that a CAF signature is overrepresented in bone metastases, as compared to lung, liver and brain metastases [37].

CAFs and OBs share a common cell-of-origin (MSC/pericyte) and some degree of marker expression, and their recruitment is induced by identical growth factors. Therefore, it is tempting to speculate that OBs are an additional, bone-specific population of CAFs and that the osteoblastic response is a tissue-specific manifestation of desmoplastic response to cancer cell invasion.

*Fscn1*, one of the 7-gene set, is the top up-regulated gene of the Core OB-BMST. Its encoded protein is critical for cell-matrix adhesion, cell interactions and cell motility. In normal adult tissue, it is expressed exclusively by dendritic cells [38]. In solid cancers, FSCN1 has been mainly associated with cancer cells and its stromal expression has been generally overlooked [39]. Here we show for the first time that, in bone metastasis, the expression of this protein is induced *de novo* in OBs and osteocytes, and in CAFs. This finding suggests *FSCN1* as a candidate biomarker for osteoblastic bone metastasis.

#### Angiogenesis

The contribution of angiogenesis to the stroma response in osteoblastic bone metastasis is substantiated by: *a)* the up-regulation of markers of ECs of endosteal, sinusoidal BM vessels, namely endomucin [40] and laminin B1 [41] and of pericytes, namely Mcam [42] and nestin [43] in the OB-BMST, *b)* the prominent representation (35%) of markers and/or effectors of EC recruitment and function among the 96 most up-regulated genes of the Core OB-BMST, *c)* the presence of VEGFA and FGF2, the most important factors in EC recruitment [44], of PDGFB, a critical recruitment factor for pericytes in normal and tumor-induced neovasculature [45] and of CTGF, a factor coordinating angiogenesis in bone [46], among the activated upstream regulators of the OB-BMST.

The finding that angiogenesis is a prominent process in osteoblastic bone metastasis is not surprising considering that, in physiological bone remodeling, it is tightly coupled to osteogenesis [40] and is required for primary and metastatic tumor growth [44]. It is also consistent with recent studies highlighting the relevance of sprouting BM neovasculature as a metastatic niche supporting cancer cell growth [47]. Nevertheless, it is surprising that the role of angiogenesis in osteoblastic bone metastasis is almost unexplored [4]. To our knowledge, only two studies have investigated the impact of anti-angiogenic therapy in mouse models of osteoblastic bone metastasis and shown inhibition of both osteoblastic response and tumor burden [48], [49]. Further investigation is warranted for determining the relevance of angiogenesis and the efficacy of anti-angiogenesis in osteoblastic bone metastasis.

*Ephb4* is an up-regulated OB-BMST gene that also emerges as one of the upstream regulators of the Core OB-BMST. The ephrinB2/Ephb4 axis is involved in coupling bone resorption to bone formation whereby OC-derived ephrinB2, by binding to Ephb4 on MSCs, induces their differentiation into OBs [50]. In line with this, interference with the ephrinB2/EphB4 axis by myeloma cells represses bone formation [51]. This axis plays also an essential role in angiogenesis [52]. Accordingly, the ephrinB2/EphB4 axis may mediate also coupling of angiogenesis and osteogenesis in osteoblastic bone metastases.

### The OB-BMST highlights amplification of SC niche components

#### Osteoinductive PCa cells amplify the HSC niche

The dominant angiogenic and osteoblastic responses found in the OB-BMST/Core OB-BMST are paralleled by up-regulation of several genes encoding proteins controlling homing/mobilization, self-renewal, dormancy or expansion of HSC and, therefore, to be considered as components of the HSC niche. Furthermore, the transcriptional components predicted to be active in the Core OB-BMST (Maz, Meis1 and Foxo4) have been implicated in both hematopoiesis [53]–[55] and angiogenesis [56]–[58].

The endosteal (OBs) and vascular (EC/pericytes) are considered the two most relevant HSC niches in the BM (reviewed in [59]) and both angiogenesis and osteogenesis are required for generation of the HSC niche [60]. Very recently, elegant studies on the spatial organization of HSC niches have revealed that the vascular and endosteal niches constitute a single structural and functional entity [41], [60]. Collectively, these studies indicate that physiological angiogenesis and osteogenesis are merged spatially and temporally to build the HSC niche. Our findings suggest that, in osteoblastic bone metastasis, the angiogenic and OB responses are also finalized to the expansion of the HSC/metastatic niche. Therefore, they seem to validate a model of cancer cell-metastatic niche interaction, whereby cancer cells not only occupy and activate, but also amplify a pre-existing SC niche, thus fuelling further tumor growth [61].

Several molecules acting at the HSC niche interface, namely, kit-ligand (Kitlg or stem cell factor, SCF) [62], growth-arrest specific 6 (GAS6) [63], annexin A2 (Anxa2) [64], chemokine (C-X-C motif) ligand 12 (CXCL12), N-cadherin (Cdh2) [65], [66] and trombospondin 1 (Thbs1) [47] have been shown to function also as components of the metastatic niche in models of osteolytic bone metastases. Only one of these genes, namely *Cdh2*, is present in the Core OB-BMST gene list. Most likely, this poor agreement reflects the fact that the HSC niche components of the Core OB-BMST are specific for the osteoblastic bone metastasis. In line with this, preliminary results from our laboratory show that the stroma transcriptome from xenograft models of osteolytic bone metastases differs substantially from the OB-OBMST and shares only 4 out of the 14 HSC genes present in the Core OB-BMST (JH, AW and MGC, unpublished).

Among the genes encoding components of the HSC niche, *Ptn* and *EphA3* are especially interesting. Ptn has been reported to be secreted by OBs [67] and ECs [68] and to be integrated in new bone matrix [67]. Here we confirm PTN expression by active OBs, but not by the BM vasculature of human samples of osteoblastic bone metastasis. While its effects on the OB lineage are still disputed [67], Ptn is known to promote tumor angiogenesis, CAF recruitment and ECM remodeling [69]. Notably, Ptn has been demonstrated to be a component of the BM vascular niche regulating HSC self-renewal and retention *in vivo* [68].

Bidirectional signaling between Eph receptors and their membrane-bound ligands, ephrins, has been shown to play a role in cell migration and adhesion, and patterning of vascular, nervous and skeletal systems [70]. Notably, Ephs/ephrins are expressed in adult, epithelial SC niches where they modulate SC function [71]. However, the involvement of EphA3 in these processes is still unexplored. EphA3 mRNA expression in cultured mouse OBs has been reported, but with no indication of its function [72]. EphA3, together with EphA2 and EphA4, has been found to be expressed by mouse BM stromal cell lines and involved in homing and mobilization of HSCs [73]. We observed EphA3 protein expression by stellate-like cells within normal, human BM and in the tumor stroma of human bone metastasis, but not in bone or other BM stromal cells. This discrepancy may be possibly due to species differences and/or *in vitro versus in vivo* detection.

Our results and the observations above propose PTN and EPHA3 as HSC niche components that may also be part of the BM metastatic niche.

#### Osteoinductive PCa cells also induce their own niche

Remarkably, 37% and 42% of the up-regulated genes of the OB-BMST and Core OB-BMST, respectively, are part of the developing prostate SC niche (UGM) signature [14]. Yet, approximately 20% of these genes are also functionally linked to the HSC niche. An example for these is *Ptn*, overexpressed in the Core OB-BMST, and component of both the HSC [13], where it modulates HSC maintenance [68], and prostate SC [14] niches, where it regulates branching morphogenesis [74]. Interestingly, binding sites for Maz, one the transcription factors predicted to be active in the Core OB-BMST, have been also found enriched in the UGM [14].

Collectively, these findings suggest that osteoinductive PCa cells not only amplify a pre-existing, BM HSC niche, but also induce *de novo* an ectopic epithelial SC niche reminiscent of the organ of origin. A similar mechanism has been demonstrated in MCa metastasis to the lung, whereby tumor initiating cells educate stromal cells of the target organ to express ECM components of the SC niche of the developing mammary gland, such as POSTN, which then become components of the metastatic niche supporting stem-like cancer cells maintenance [75].

The combinatorial niche association of HSC and epithelial SC niches may create a “soil” suitable for stem-like cancer cell growth, but hostile for HSCs, thus explaining the hematopoietic aplasia (myelophthisis) occurring in bone metastasis [76] and in myeloproliferative neoplasia [77]. It may also correspond to the growth requirements specific for osteoinductive PCa cells. Furthermore, the capacity to induce *de novo* prostate epithelial SC niche components in the BM, but not in other tissues, as shown here, could also be a mechanism explaining the osteotropism of PCa cells.

### The OB-BMST also contains gene signatures not unique to osteoblastic bone metastasis

#### Inflammatory/wound healing/desmoplastic response

A first fraction of the OB-BMST contains genes of inflammation/wound healing/desmoplasia-related signatures. This is not surprising in view of the similarities between wound healing and cancer [78] and of the persistent activation of wound-healing and inflammatory programs in tumors [79]. Clearly, genes contained in this fraction are not cancer-restricted, thus limiting their use as cancer biomarkers. POSTN, one of these genes, has been proposed as a serum biomarker for bone metastasis [80]. However, its validity in this regard should be proven *versus* a clinical scenario of inflammation.

#### “Universal” stroma response to cancer

A second fraction of the OB-BMST contains genes shared by cancers with no or low propensity to metastasize to bone. This component is species, tissue and cancer cell type independent and thus seems to be a “universal” stroma response to cancer cells. Accordingly, genes part of this OB-BMST component could represent general biomarkers of cancer, but not of a specific cancer type.

### Stroma osteomimicry in primary tumor as possible determinant of cancer osteotropism

An additional fraction of the OB-BMST contains genes shared by primary PCa and MCa known for their high propensity to metastasize to bone (osteotropism).

It has been hypothesized that the osteotropism of PCa is conferred by the capacity of PCa cells to aberrantly express OB-restricted proteins, such as osteopontin and osteocalcin. This phenomenon, named osteomimicry, would allow the cancer cells to thrive specifically in the bone microenvironment [81]. Recently, it has been suggested that osteomimicry by the stroma of the primary tumor, rather than by cancer cells, may also explain the osteotropism of MCa [37]. Our results may extend this notion to PCa and expand the list of molecules potentially involved.

*Mcam*, also known as *Cd146*, is of special interest in this regard. It is a cell adhesion molecule expressed at the intercellular junction of ECs and in activated T lymphocytes. A homophilic MCAM interaction between these cells mediates lymphocyte trafficking at inflammatory sites [82]. MCAM expression in human BM marks specifically pericytes of the sinusoids [42], an essential cell population of the HSC niche [43]. MCAM expression by cancer cells has been reported in most cancer types [83]. Increased stromal expression of MCAM parallels metastatic potential in osteotropic cancers, including PCa and MCa [84], [85]. Importantly, *MCAM* is present in a gene signature predicting bone metastatic progression in MCa [86]. Therefore, MCAM-mediated homophilic interaction between cancer and BM stromal cells may represent a possible molecular mechanism of osteotropism.

The fraction of the OB-BMST common to the stroma response of primary PCa and MCa may contain a gene signature that may predict, similarly to MCAM, disease progression as bone metastasis. Therefore, it warrants to be verified in gene expression datasets, once available, of primary PCa from patients with or without bone metastatic relapse.

### Translational significance

The OB-BMST/Core OB-BMST will serve as a reference list of physiological SC niche components to be validated as novel components of the BM/B metastatic niche. Besides their mechanistic significance, these molecules may represent additional therapeutic targets allowing interference with cancer cell homing, survival and growth in the BM/B. Indeed, it has been shown that cancer cells can be mobilized from the HSC niche into the blood by using HSC mobilizing agents [87]. Furthermore, anti-angiogenesis, by limiting the size of the SC/metastatic niches, may also interfere with survival and growth of osteoinductive PCa cells in the BM/B.

Modifications of the cancer-associated plasma proteome seem to be predominantly derived from the tumor microenvironment [88]. Therefore, the Core OB-BMST could also provide novel stroma-derived, serum biomarkers for the detection of bone metastatic cancer progression.

## Materials and Methods

### Ethics statement

The Ethical Committee of the Canton of Bern, Switzerland, approved the overall study protocol and tissue collection from patients (Nr 06/03). A written informed consent was obtained from each patient.

The Committee for Animal Experimentation and the Veterinary Authorities of the Canton of Bern, Switzerland approved the experimental animal protocols, anesthesia, surgical procedures and post-surgical analgesia (Permit Number: 15/07 and 6/10). Mice were housed in individual ventilated cages in strict accordance to the Swiss Guidelines for the Care and Use of Laboratory Animals. Autoclaved water and sterile mouse chow were provided *ad libitum*. For surgical manipulation, mice were anesthetized with a cocktail of medetomidin (1 mg/kg body weight), midazolam (10 mg/kg) and fentanyl (0.1 mg/kg) [34]. Post-operative analgesia with buprenorphine (0.1 mg/kg) was performed for 3 days following surgical intervention. Animals xenografted with human cancer cells were carefully monitored for signs of pain, distress and loss of body weight. Development of bone lesions was followed by radiography at two weeks intervals for VCaP and C4-2B and at weekly intervals for PC-3 cells. The size of subcutaneous and orthotopic tumors never exceeded 200 mm^3^. At the experimental endpoint mice were sacrificed by CO_2_ euthanasia.

### Cell culture

The PCa cell line C4-2B [89] and the luciferase-transfected C4-2B*luc* [34] were grown in T-medium [89], and the PCa cell line VCaP (kindly donated by Dr. K. Pienta, University of Michigan, Ann Arbor) [90] in RPMI 1640 medium (Biochrom AG, Berlin, Germany). The PCa cell line PC-3 (ATCC CRL1435) was grown in DMEM and the prostate epithelial cell line Ep156T (kindly donated by Dr. V. Rotter, Department of Molecular Cell Biology, Weizmann Institute of Science, Rehovot, Israel) [91] in modified MCDB-153 medium (WKS Diagnostics, Frankfurt am Main, Germany). All media were supplemented with 10% fetal bovine serum (Oxoid, Pratteln, Switzerland).

### Xenografts

For intra-osseous xenografts, PCa cells with low/medium (C4-2B cell line) or high (VCaP cell line) capacity to induce an osteoblast response (osteoinductive cancer cells), and PCa cells (PC-3 cell line) with high capacity to induce an osteoclast reaction (pro-osteolytic cancer cells) and immortalized, non-tumorigenic human prostate epithelial Ep156T cells were inoculated in the BM cavity of the left tibia of male CB17 SCID mice [34]. Sham-operated animals (sham) and animals not subjected to surgery (intact) were used as controls. Development of bone lesions was monitored by radiography. Mice xenografted with VCaP and Ep156T cells were sacrificed after 6 weeks, and those inoculated with C4-2B after 8 weeks. PC-3 xenografts and their Ep156T controls animals were sacrificed after 33 days. Sham and intact animals were sacrificed at the 3 time points above. Xenografted and control tibiae were used either for RNA isolation or immunohistochemistry.

VCaP cells were used for orthotopic (intra-prostate) implantation, while C4-2B*luc* and VCaP cells were used for subcutaneous implantation. For both types of xenografts, a suspension of 10E06 cells was mixed with collagen (Collagen Type I from rat tail, BD Biosciences, Allschwil, Switzerland). Prostates implanted with collagen pellets and dermal tissue served as control. Subcutaneous tumors were excised after 9 weeks and orthotopic tumors after 10 weeks. For more details see **S1 File**.

### RNA extraction

RNA was extracted from cultured C4-2B and VCaP cells, from C4-2B, VCaP, Ep156T and PC-3 xenografted bones, from sham-operated and intact bones, and from sub-cutaneous and orthotopic xenografts using an RNeasy isolation kit (Qiagen, Hombrechtikon, Switzerland). The ratio of human to mouse RNA in the xenograft samples was determined by measuring 18S and both mouse and human β2-microglobulin, hypoxanthine phosphoribosyltransferase 1 and actin beta expression with RT-qPCR. The human to mouse ratio was 1∶1 for C4-2B, 1∶5 for VCaP and 1∶9 for PC-3 intra-osseous xenografts, 4∶1 for C4-2B and VCaP subcutaneous xenografts and 1∶2 for VCaP orthotopic xenografts. RNA quality was assessed using Agilent 2100 Bioanalyzer (Agilent Technologies, Basel, Switzerland).

### Microarray hybridization and data analysis

Labeled cRNA was prepared according to Affymetrix protocols and hybridized to Mouse Genome 430A 2.0 arrays (Affymetrix Ltd., High Wycombe, UK). The hybridization was performed with 30 µg cRNA for VCaP and Ep156T xenografts, and for intact, sham bones. Double the amount of cRNA was used for the C4-2B xenografts in order to correct for the high human RNA content in these xenografts. Three replicates were performed for all experimental groups except sham-operated bones and Ep156T xenografts, which were done in duplicates. Quality control of the microarray data was performed using RReportGenerator [92] and confirmed that all arrays used in the study were of good and consistent quality. Raw data are available on GEO (GSE22813). We computed statistical significance using the standard moderated t-test approach of the limma package [93] and computed local false discovery rates (FDR) [94] to adjust statistical significance to multiple-testing.

To reduce the impact of cross-species hybridization on gene expression signals, we re-defined probe-sets in order to use only probes considered mouse-specific (for a detailed description of the identification of cross-hybridising probe-sets see **S1 File**. After exclusion of cross-hybridizing probe-sets, the 3 control groups were analyzed for gene expression changes using a FDR of 0.2. Only a single gene, namely MMP12, was differentially expressed between intact bones and sham-operated/Ep156T-xenografted bones.

Subsequently, differential gene expression between xenografted bones and sham-operated bones was calculated. The different human to mouse RNA ratio for the different samples was taken into account when selecting highly stringent thresholds in the statistical testing. Two different FDR thresholds were selected for VCaP (FDR≤3E-05) and C4-2B (FDR≤1E-05) due to different distribution of values.

### Reverse transcription quantitative PCR (RT-qPCR)

RNA was reverse transcribed with M-MLV-RT (Promega, Wallisellen, Switzerland) and random primers (Roche Diagnostics, Rotkreuz, Switzerland) in the presence of a RNase inhibitor (Promega). mRNA expression was measured by RT-qPCR using ABI Prism Sequence Detection Systems (Applied Biosystems, Rotkreuz, Switzerland). The mouse and human specific gene expression assays are listed in the **S1 File**. RNA expression data were analyzed with two-tailed, unpaired t-test using GraphPad Prism version 6.0 (GraphPad Software Inc., La Jolla, USA).

### Immunohistochemistry

Immunohistochemical staining was performed on deparaffinized tissue sections with the primary antibodies listed in the **S1 File**. Antibodies were detected using horseradish peroxidase-conjugated biotin-streptavidin (GE Healthcare, Glattbrugg, Switzerland) or EnVision (Dako, Baar, Switzerland) systems. 3-Amino-9-ethyl-carbazole (AEC, Sigma) was used as a chromogen. Sections were counterstained with hematoxilin.

Samples of normal bone were obtained from patients with coxarthrosis. PCa bone metastasis samples were obtained from iliac crest and femur, while MCa bone metastasis samples were obtained from the humerus. Samples of normal prostate tissue were obtained from cystoprostatectomy for bladder cancer. Samples of PCa of Gleason grades 3/4 were obtained from radical prostatectomy.

### Validation of the stroma specificity of the OB-BMST

We confirmed the absence of 25 accepted pan-epithelial and/or prostate epithelial cell-specific markers in the OB-BMST. Furthermore, a literature review of 15 selected genes strongly up-regulated in the OB-BMST confirmed their stromal origin. The differential expression and stroma specificity of 7 representative genes was validated by RT-qPCR using mouse-specific probes. We also confirmed the stromal expression of two proteins (Aspn and Postn) encoded by up-regulated genes of the OB-BMST. More details concerning the stroma specificity of the OB-BMST are available in the **S1 File**.

Taken together these results show the reliability of our approach to analyze specifically the stroma compartment.

### Curation strategy

To obtain a bone-specific stroma response signature we adopted the following curation strategy (**Fig. 1A** **)**. First, we subtracted from the OB-BMST desmoplastic [15], wound healing [95] and inflammatory [16], [18], [19] response signatures. Subtraction of the 85 overlapping genes generated a “Curated 1” OB-BMST list. Next, we subtracted stroma signatures derived from cancers that do not or rarely metastasize to bone, namely gastric [20], pancreatic [21], [22], colorectal [23] and esophageal [24], [25] cancers. Subtraction of the 79 overlapping genes generated a “Curated 2” OB-BMST. From this we further subtracted stroma gene expression signatures derived from primary PCa [29]–[33] and MCa [12], [26]–[29]. After subtraction of the 88 overlapping genes, we obtained the Core OB-BMST, which represents the specific BM/B response to osteoinductive PCa cells. A more detailed description of the curation strategy is available in the **S1 File**.

### Identification of key biological processes

The OB-BMST and Core OB-BMST common to VCaP and C4-2B (“common” OB-BMST/Core OB-BMST) were analyzed for enriched gene ontology (GO) terms (DAVID 6.7; FDR<0.5) and functional protein networks (STRING, 9.05; confidence score = 0.4). The interaction partners highlighted by the STRING analysis were assigned to biological processes according to BioGPS platform (http://biogps.org/).

The upstream regulators of the “common” OB-BMST and Core OB-BMST were predicted by using default options of the Ingenuity Pathway Analysis software.

Genes of the “common” Core OB-BMST were analyzed for the over-representation of sequence motifs in their promoters within 2-kilobases of the transcription start site. Enrichment was calculated against a matched number of randomly selected genes and represented as *p* values versus this random set and also as an enrichment factor, which is the frequency of the transcription factor binding motif within the gene cluster versus its frequency within the background gene set as previously reported [14]. We report transcription factor motif using enrichment factors of 1.2–1.5 and *p* values <10E-05. The motif database used for this analysis is supplied by TRANSFAC [96]. Results were further validated using the accessible web-based tool called GeneCodis (http://genecodis.cnb.csic.es/) [97].

## Supporting Information

S1 Figure **The OB-BMST overlaps with myoepithelial/myofibroblast signature and, to a lesser extent, with fibroblast and endothelial cell signatures.** Venn diagrams and tables showing overlap of the up-regulated genes of the OB-BMST (human orthologs) with gene signatures previously derived from specific stromal cell populations from normal mammary tissue, *in situ* (ductal carcinoma *in situ*, DCIS) and invasive MCa (Allinen *et al*. 2004). **A**. Myoepithelial/myofibroblasts. **B**. Fibroblasts. **C**. Endothelial cells. **D**. Leukocytes.
(TIF)

S2 Figure **A fraction of the OB-BMST is not specific for the BM/B response to osteoinductive PCa cells.** Relative expression levels of *Postn, Aspn, Sparcl1, Mcam, Pdgfrb, Fscn1* and *Pmepa1* mRNA in intra-osseous, orthotopic and ectopic xenografts. **A.** VCaP intra-osseous xenografts (grey, *n* = 3) and corresponding sham-operated bones (white, *n* = 3) and C4-2B xenografts (black, *n* = 4) and corresponding sham-operated bones (white, *n* = 3). Values are shown as fold-change (mean ± SD) relative to contralateral bones. **B.** VCaP orthotopic xenografts (grey, *n* = 5) and sham (white, *n* = 4). Values are shown as fold-change (mean ± SD) relative to intact prostate. **C.** VCaP (grey, *n* = 3) and C4-2B (black, *n* = 5) subcutaneous xenografts. Values are shown as fold-change (mean ± SD) relative to intact skin. **D.** PC-3 intra-osseous xenografts (light grey, *n* = 6) and sham (white, *n* = 4). Values are shown as fold-change (mean ± SD) relative to contralateral bones (*n* = 3–4). *, *P*<0.01; **, *P*<0.001; ***, *P*<0.0001; ****, *P*<0.0001, ns  =  not statistically significant. Abbreviations: *Postn,* periostin; *Aspn,* asporin; *Sparcl1,* SPARC-like 1; *Mcam,* melanoma cell adhesion molecule; *Pdgfrb,* platelet derived growth factor receptor beta; *Fscn1,* fascin homolog 1; *Pmepa1,* prostate transmembrane protein, androgen induced 1.
(TIF)

S3 Figure **Periostin and asporin expression is induced in the stroma of human bone metastatic PCa and MCa and of primary PCa.** Immunohistochemical detection of POSTN (**A**, **C**, **E**, **G** and **I**) and ASPN (**B**, **D**, **F**, **H** and **J**) in normal bone (**A** and **B**), in PCa bone metastasis (**C** and **D**), in MCa bone metastasis (**E** and **F**), in normal prostate (**G** and **H**) and in primary PCa (**I** and J). Normal bone and hematopoietic marrow (**A**) are lacking POSTN immunoreactivity. In contrast, in PCa (**C**) and MCa (**E**) bone metastases, myofibroblasts surrounding areas of cancer cell growth are POSTN-positive. OBs, osteocytes, OCs and cancer cells are negative. Normal prostate (**G**) is devoid of POSTN immunoreactivity both in the stroma and epithelial compartment. In contrast, in PCa (**I**) strong POSTN immunoreactivity is found in myofibroblasts over the entire tumor stroma, while cancer cells are negative. The myofibroblast identity of the POSTN-immunoreactive cells was confirmed in PCa by co-staining with α-smooth muscle actin (not shown). In normal bone (**B**), ASPN immunoreactivity is detected in OBs at sites of active bone formation, while lining cells, osteocytes and OCs are negative. Spindle-like cells within the hematopoietic marrow are also positive. In PCa (**D**) and MCa (**F**) bone metastases, strong ASPN immunoreactivity is detected in active OBs, and additionally in lining cells, osteocytes, and OB precursors. Stromal cells within areas of cancer cells are also ASPN-positive whereas cancer cells are ASPN-negative. In normal prostate (**H**) ASPN immunoreactivity is found in fibroblast-like cells and EC of small vessels, but not in epithelial cells. In the prostate, ASPN expression is also detected in cells, identified, in sequential sections, as neuroendocrine by expression of chromogranin-A and synaptophysin and in Schwann cells (not shown). In PCa (**J**) the number of ASPN-positive, fibroblast-like cells is increased. In some specimens rare PCa cells are stained for ASPN (not shown). Insets represent a higher magnification of selected areas. Scale bar = 50* µ*m. Abbreviations: POSTN, periostin; ASPN, asporin.
(TIF)

S1 Table **Differentially expressed genes of the OB-BMST: (A) C4-2B xenografts (FDR≤1E-05), (B) VCaP xenografts (FDR≤3E-05) and (C) common to both C4-2B and VCaP.**
(XLS)

S2 Table **Differentially expressed genes of the Core OB-BMST: (A) C4-2B xenografts (FDR≤1E-05), (B) VCaP xenografts (FDR≤3E-05) and (C) common to both C4-2B and VCaP.**
(XLS)

S3 Table **Activated upstream regulators of the common OB-BMST.**
(XLS)

S4 Table **Literature survey of up-regulated Core OB-BMST genes.**
(DOC)

S5 Table **Gene list of HSC niche (Charbord** ***et al.*** **) and prostate SC niche (Blum** ***et al.*** **).**
(XLSX)

S6 Table **Overlapping genes of the OB-BMST with wound, desmoplastic response, non-osteotropic and osteotropic gene lists.**
(XLS)

S1 File **This file contains an extended version of the Material and Methods, including references, one Figure and 11 Tables.**
(ZIP)
