# Supplementary figures and images for: The Molecular Signature of the Stroma Response in Prostate Cancer-Induced Osteoblastic Bone Metastasis Highlights Expansion of Hematopoietic and Prostate Epithelial Stem Cell Niches
Source: PLoS One. 2014 Dec 8;9(12):e114530. doi: 10.1371/journal.pone.0114530 (PMC4259356; doi:10.1371/journal.pone.0114530)

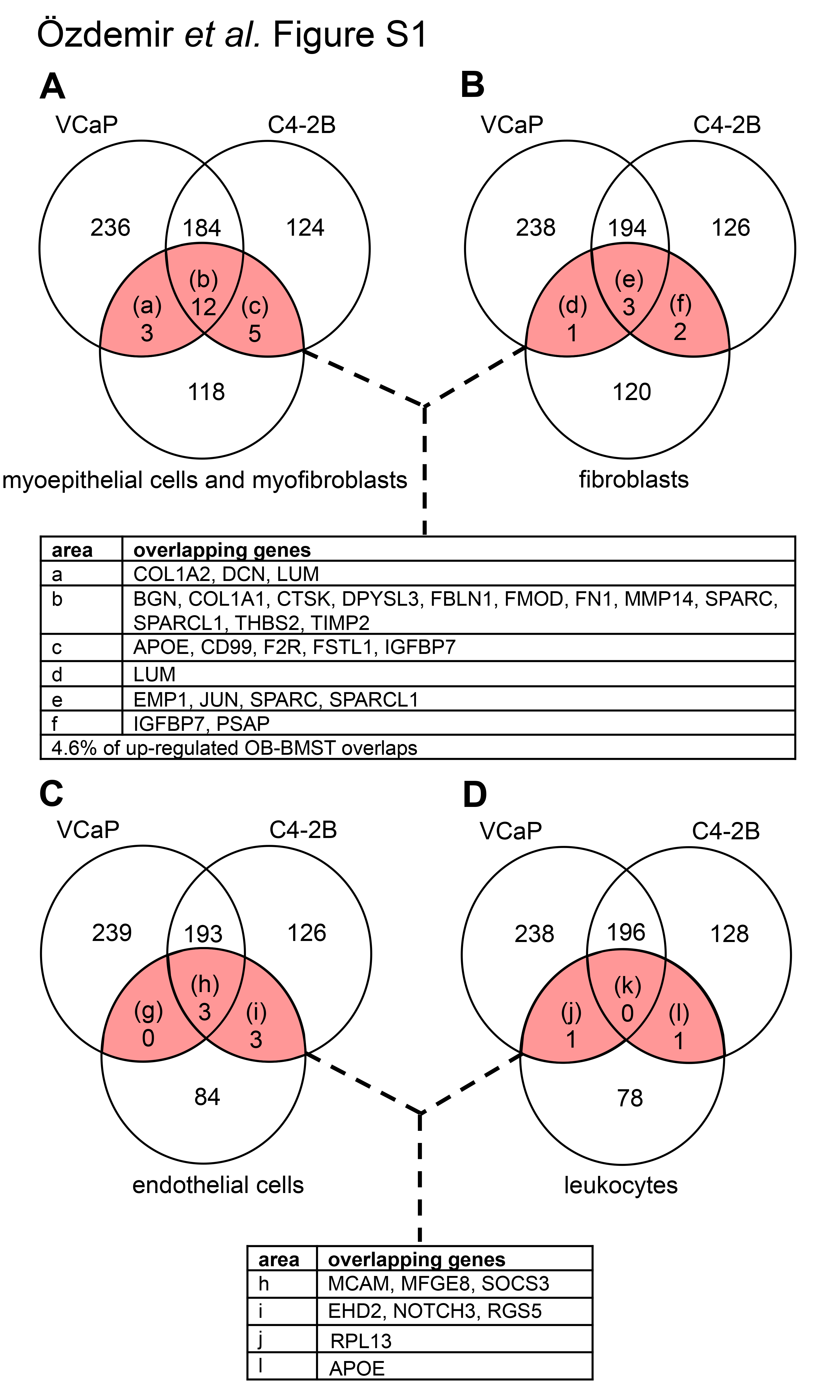

Supplement: S1 Figure — The OB-BMST overlaps with myoepithelial/myofibroblast signature and, to a lesser extent, with fibroblast and endothelial cell signatures. Venn diagrams and tables showing overlap of the up-regulated genes of the OB-BMST (human orthologs) with gene signatures previously derived from specific stromal cell populations from normal mammary tissue, in situ (ductal carcinoma in situ, DCIS) and invasive MCa (Allinen et al. 2004). A. Myoepithelial/myofibroblasts. B. Fibroblasts. C. Endothelial cells. D. Leukocytes. (TIF) [file pone.0114530.s001.tif]

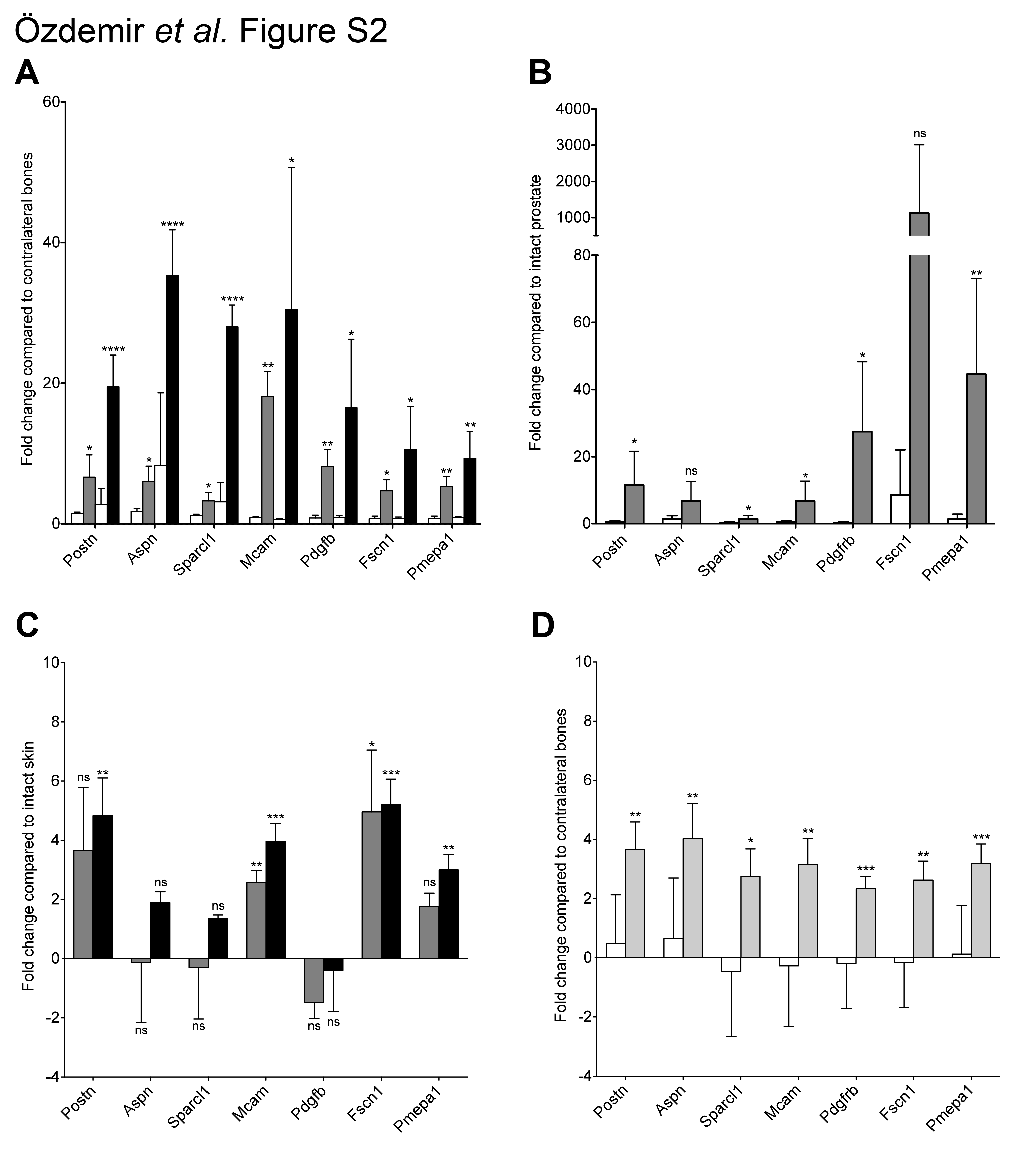

Supplement: S2 Figure — A fraction of the OB-BMST is not specific for the BM/B response to osteoinductive PCa cells. Relative expression levels of Postn, Aspn, Sparcl1, Mcam, Pdgfrb, Fscn1 and Pmepa1 mRNA in intra-osseous, orthotopic and ectopic xenografts. A. VCaP intra-osseous xenografts (grey, n = 3) and corresponding sham-operated bones (white, n = 3) and C4-2B xenografts (black, n = 4) and corresponding sham-operated bones (white, n = 3). Values are shown as fold-change (mean ± SD) relative to contralateral bones. B. VCaP orthotopic xenografts (grey, n = 5) and sham (white, n = 4). Values are shown as fold-change (mean ± SD) relative to intact prostate. C. VCaP (grey, n = 3) and C4-2B (black, n = 5) subcutaneous xenografts. Values are shown as fold-change (mean ± SD) relative to intact skin. D. PC-3 intra-osseous xenografts (light grey, n = 6) and sham (white, n = 4). Values are shown as fold-change (mean ± SD) relative to contralateral bones (n = 3–4). *, P<0.01; **, P<0.001; ***, P<0.0001; ****, P<0.0001, ns = not statistically significant. Abbreviations: Postn, periostin; Aspn, asporin; Sparcl1, SPARC-like 1; Mcam, melanoma cell adhesion molecule; Pdgfrb, platelet derived growth factor receptor beta; Fscn1, fascin homolog 1; Pmepa1, prostate transmembrane protein, androgen induced 1. (TIF) [file pone.0114530.s002.tif]

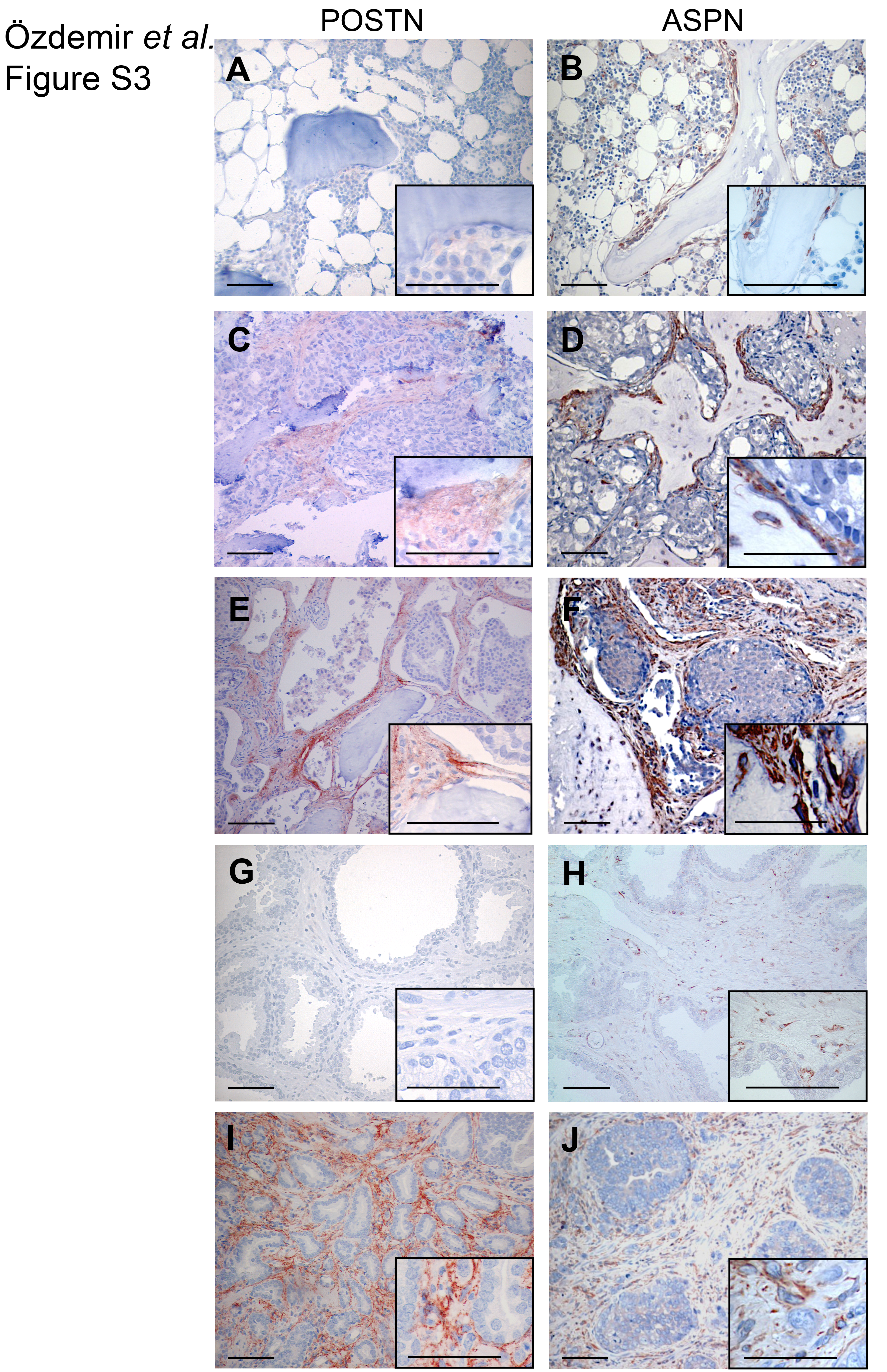

Supplement: S3 Figure — Periostin and asporin expression is induced in the stroma of human bone metastatic PCa and MCa and of primary PCa. Immunohistochemical detection of POSTN (A, C, E, G and I) and ASPN (B, D, F, H and J) in normal bone (A and B), in PCa bone metastasis (C and D), in MCa bone metastasis (E and F), in normal prostate (G and H) and in primary PCa (I and J). Normal bone and hematopoietic marrow (A) are lacking POSTN immunoreactivity. In contrast, in PCa (C) and MCa (E) bone metastases, myofibroblasts surrounding areas of cancer cell growth are POSTN-positive. OBs, osteocytes, OCs and cancer cells are negative. Normal prostate (G) is devoid of POSTN immunoreactivity both in the stroma and epithelial compartment. In contrast, in PCa (I) strong POSTN immunoreactivity is found in myofibroblasts over the entire tumor stroma, while cancer cells are negative. The myofibroblast identity of the POSTN-immunoreactive cells was confirmed in PCa by co-staining with α-smooth muscle actin (not shown). In normal bone (B), ASPN immunoreactivity is detected in OBs at sites of active bone formation, while lining cells, osteocytes and OCs are negative. Spindle-like cells within the hematopoietic marrow are also positive. In PCa (D) and MCa (F) bone metastases, strong ASPN immunoreactivity is detected in active OBs, and additionally in lining cells, osteocytes, and OB precursors. Stromal cells within areas of cancer cells are also ASPN-positive whereas cancer cells are ASPN-negative. In normal prostate (H) ASPN immunoreactivity is found in fibroblast-like cells and EC of small vessels, but not in epithelial cells. In the prostate, ASPN expression is also detected in cells, identified, in sequential sections, as neuroendocrine by expression of chromogranin-A and synaptophysin and in Schwann cells (not shown). In PCa (J) the number of ASPN-positive, fibroblast-like cells is increased. In some specimens rare PCa cells are stained for ASPN (not shown). Insets represent a higher magnification of se [file pone.0114530.s003.tif]
