# Supplementary material for: The Molecular Signature of the Stroma Response in Prostate Cancer-Induced Osteoblastic Bone Metastasis Highlights Expansion of Hematopoietic and Prostate Epithelial Stem Cell Niches
Source: PLoS One. 2014 Dec 8;9(12):e114530. doi: 10.1371/journal.pone.0114530 (PMC4259356; doi:10.1371/journal.pone.0114530)
Supplement: S4 Table — Literature survey of up-regulated Core OB-BMST genes. (DOC) [file pone.0114530.s007.doc]

**Supporting Table S4.** Literature survey of up-regulated Core OB-BMST genes.

| **Gene symbol** | **Gene name** | **Fold change** **VCaP / C4-2B** | **FDR** **VCaP / C4-2B** | **Functions** | **References** |
| --- | --- | --- | --- | --- | --- |
| **Osteoblast marker, recruitment and/or function** | | | | | |
| *Abca1* | ATP-binding cassette, sub-family A (ABC1), member 1 | 3.32 / 4.05 | 6.73E-06 / 4.65E-06 | Regulates RANKL:OPG ration in osteoblasts | Kleyer *et al*, 2012[1] |
| *Alpl* | Alkaline phosphatase, liver/bone/kidney | 9.45 / 2.52 | 3.59E-06 / 1.21E-03 | Involved in mineralization | Narisawa *et al.*, 2013[2] |
| *Bmp1* | Bone morphogenetic protein 1 | 9.11 / 4.29 | 1.34E-06 / 3.84E-06 | Plays a role in osteoblast differentiation and bone formation | Grgurevic *et al.*, 2011[3] |
| *Cd200* | Cd200 antigen | 4.41 / 2.83 | 1.20E-06 / 5.90E-06 | Expressed on osteoblast progenitor cells; increased endogenous expression leads to increased OPG:RANKL ratios and bone nodule growth | Lee *et al.*, 2006[4] |
| *Cdh2* | Cadherin 2 | 5.94 / 1.86 | 1.68E-05 / 1.81E-02 | Regulates osteoblast differentiation | Ferrari *et al*., 2000[5] |
| *Cdo1* | Cysteine dioxygenase type 1 | 7.57 / - | 1.20E-06 | Runx-2 target and osteoblast marker/function | Hecht *et al.,* 2007[6] |
| *Cgref1* | Cell growth regulator with EF-hand domain 1 | 12.62 / - | 1.72E-06 | Runx-2 target and osteoblast marker/function | Hecht *et al.,* 2007[6] |
| *Creb3l1* | cAMP responsive element binding protein 3-like 1 | 11.21 / 3.38 | 1.48E-06 / 8.36E-05 | Expressed in osteoblasts; regulates transcription of Col1a1 | Murakami *et al.*, 2009[7] |
| *Enpep* | Glutamyl aminopeptidase | 2.42 / 4.98 | 2.04E-04 / 1.75E-06 | Marker of common skeletal progenitor cells | Chan *et al.*, 2013[8] |
| *EphA3* | Eph receptor A3 | 17.53 / 14.39 | 1.20E-06 / 1.51E-06 | Runx-2 target and osteoblast marker/function | Hecht *et al.,* 2007[6] |
| *Ephb4* | Eph receptor B4 | 3.10 / 3.37 | 1.17E-05 / 3.84E-06 | Receptor on osteoblasts; overexpression in mice leads to increased bone formation | Martin *et al.*, 2010[9] |
| *Hoxa3* | Homeo box A3 | 5.16 / 4.08 | 1.48E-06 / 1.96E-06 | Function in bone mineralization | Kovacs *et al.*, 2001[10] |
| *Itm2a* | Integral membrane protein 2A | - / 5.15 | 5.90E-06 | Osteoblast marker | Tuckermann *et al*., 2000[11] |
| *Ltbp3* | Latent transforming growth factor beta binding protein 3 | 3.19 / 2.47 | 1.48E-06 / 3.88E-06 | Involved in osteogenic differentiation | Koli *et al.*, 2008[12] |
| *Ltbp4* | Latent transforming growth factor beta binding protein 4 | 3.37 / 7.37 | 6.73E-06 / 3.08E-07 | Expressed in differentiating MSC-derived osteoblasts | Mukherjee *et al.*, 2012[13] |
| *Mmp23* | Matrix metallopeptidase 23 | 5.84 / 5.39 | 1.17E-05 / 8.20E-06 | Expressed in osteoblasts; suggested role in bone formation | Clancy *et al.*, 2003[14] |
| *Mrc2* | Mannose receptor C, type 2 | 4.67 / 3.72 | 7.11E-06 / 3.84E-06 | Expressed in osteoblasts, dysregulated in bone metastasis | Caley *et al*., 2011[15] |
| *Nab2* | NGFI-A binding protein 2 (EGR1 binding protein 2) | 4.09 / 2.69 | 1.48E-06 / 8.20E-06 | Osteoblast recruitment | Chandra *et al*., 2013[16] |
| *Nos3* | Nitric oxide synthase 3, (endothelial cell) | 3.12 / 3.22 | 6.73E-06 / 2.77E-06 | Regulates osteoblast function | Aguirre *et al.*, 2001[17] |
| *Plat* | Plasminogen activator, tissue | 4.22 / 14.91 | 1.17E-05 / 2.24E-07 | Induced in osteoblasts upon 1.25 vitamin D3 stimulation | Tarroni *et al.*, 2012[18] |
| *Plod1* | Procollagen-lysine, 2-oxoglutarate 5-dioxygenase 1 | 3.47 / 3.28 | 1.62E-05 / 8.20E-06 | Induced during osteoblastogenesis | Bustos-Valenzuela *et al.*, 2011[19] |
| *Pltp* | Phospholipid Transfer Protein | 4.30 / 5.44 | 9.64E-07 / 3.83E-07 | Runx-2 target and osteoblast marker/function and putative role in MSC differentiation into osteoblast/adipocyte | Hecht *et al.,* 2007[6], Scheideler *et al*., 2008[20] |
| *Ptn* | Pleiotrophin | 8.55 / 16.02 | 1.20E-06 / 3.08E-07 | Regulates bone morphogenic protein-induced ectopic osteogenesis | Sato *et al.*, 2002[21] |
| *Ramp2* | Receptor (calcitonin) activity modifying protein 2 | 3.40 / 3.65 | 6.73E-06 / 2.74E-06 | Mediates osteoblast recruitment induced by adrenomedullin | Naot *et al.,* 2001[22], Uzan *et al*., 2004[23] |
| *Ramp3* | Similar to receptor activity modifying protein 3 | 4.09 / 6.35 | 1.20E-06 / 2.24E-07 | PTH induces Ramp3 expression in osteoblasts | Phelps *et al.*, 2005[24], Uzan *et al*., 2004[23] |
| *Slc39a13* | Solute carrier family 39 (metal ion transporter), member 13 | 3.17 / 2.39 | 1.96E-06 / 8.20E-06 | Involved in the maturation of osteoblasts | Fukada *et al.*, 2008[25] |
| *Slco2a1* | solute carrier organic anion transporter family, member 2A1 | 3.16 / 4.34 | 6.73E-06 / 2.68E-06 | Negative regulator of osteoblast recruitment | Zhang *et al*., 2012[26], Seifert *et al*., 2012[27] |
| *Smpd3* | Sphingomyelin phosphodiesterase 3, neutral | 6.26 / 1.61 | 1.17E-05 / 5.39E-02 | Runx2 regulated gene; suggested to play a role in bone mineralization | Chae *et al.*, 2009[28] |
| *Sp7* | Sp7 transcription factor 7 | 6.17 / 1.36 | 1.20E-06 / 6.17E-02 | Promotes osteoblast maturation | Gao et al., 2004[29] |
| *Tgfbr2* | Transforming growth factor, beta receptor II | 3.18 / 2.98 | 1.30E-05 / 8.20E-06 | Regulates osteoblast differentiation | Seo and Serra, 2009[30] |
| *Tmem119* | Transmembrane protein 119 | 9.17 / 3.22 | 6.73E-06 / 2.85E-04 | Regulates osteoblastogenesis | Mizuhashi *et al.*, 2012[31], Hecht *et al.,* 2007 |
| *Wwtr1* | WW domain containing transcription regulator 1 | 3.88 / 4.21 | 5.81E-06 / 1.98E-06 | Promotes osteoblast lineage commitment | Byun *et al.*, 2012[32] |
| **Osteoclast marker, recruitment and/or function** | | | | | |
| *Abca1* | ATP-binding cassette, sub-family A (ABC1), member 1 | 3.32 / 4.05 | 6.73E-06 / 4.65E-06 | Inhibits osteoclasts recruitment | Kleyer *et al*, 2012[1] |
| *Cd109* | Cd109 antigen | 12.62 / 4.84 | 1.20E-06 / 8.20E-06 | Promotes osteoclast recruitment | Wang *et al.*, 2013[33] |
| *Cd200* | Cd200 antigen | 4.41 / 2.83 | 1.20E-06 / 5.90E-06 | Regulates osteoclast fusion; inhibits osteoclast recruitment | Cui *et al.*, 2007[34], Lee *et al.*, 2006[4], Varin *et al*., 2013[35] |
| *Nab2* | Ngfi-A binding protein 2 | 4.09 / 2.69 | 1.48E-06 / 8.20E-06 | Inhibits osteoclast differentiation | Kim *et al.*, 2012[36] |
| *Ocstamp* | Osteoclast stimulatory transmembrane protein | 8.58 / 2.09 | 1.10E-06 / 5.68E-04 | Promotes osteoclast fusion | Xing *et al.*, 2012[37] |
| *Serpine2* | Serine (or cysteine) peptidase inhibitor, clade E, member 2 | 3.29 / 2.42 | 1.20E-06 / 3.84E-06 | Postulated inhibitor of osteoclast recruitment and activity | Yang JN *et al*., 1997[38], Coudert *et al*., 2014[39] |
| **Endothelial cells markers, recruitment and/or function** | | | | | |
| *Abca1* | ATP-binding cassette, sub-family A (ABC1), member 1 | 3.32 / 4.05 | 6.73E-06 / 4.65E-06 | Expressed in EC | Vaisman *et al*., 2012[40] |
| *Bmp1* | Bone morphogenetic protein 1 | 9.11 / 4.29 | 1.34E-06 / 3.84E-06 | Indirect anti-angiogenic activity | Ge *et al*., 2007[41], Gonzalez *et al*., 2005[42] |
| *Cav2* | Caveolin 2 | 3.06 / 2.94 | 1.17E-05 / 6.87E-06 | Expressed in EC | Sowa, 2012[43] |
| *Cd109* | Cd109 antigen | 12.62 / 4.84 | 1.20E-06 / 8.20E-06 | Marker of EC | Hwang *et al*., 2013[44] |
| *Cd200* | Cd200 antigen | 4.41 / 2.83 | 1.20E-06 / 5.90E-06 | Expressed on EC, mediates T cell adhesion | Ko *et al*., 2009[45] |
| *Cdh2* | Cadherin 2 | 5.94 / 1.86 | 1.68E-05 / 1.81E-02 | Induces EC proliferation, stabilizes EC-mural cells interactions | Luo *et al*., 2005, Gerhardt *et al*., 2003 |
| *Colec12* | Collectin sub-family member 12 | - / 2.69 | - / 8.20E-06 | Scavenger receptor, expressed on EC | Ohtani *et al*., 2001[46] |
| *Creb3l1* | cAMP responsive element binding protein 3-like 1 | 11.21 / 3.38 | 1.48E-06 / 8.36E-05 | Regulates VEGFA transcription | Miyagi *et al*., 2013[47] |
| *Cxcl16* | Chemokine (C-X-C motif) ligand 16 | 4.44 / 8.62 | 1.02E-04 / 4.97E-06 | Promotes angiogenesis | Smith *et al.*, 2012[48] |
| *Ece1* | Endothelin converting enzyme 1 | 3.56 / 3.23 | 7.64E-06 / 6.07E-06 | Expressed in EC | Zorrilla *et al*., 2010[49], Lopez-Ongil *et al*., 2005[50] |
| *Elk3* | ELK3, ETS-domain protein (SRF accessory protein 2) | 3.20 / 3.58 | 6.73E-06 / 1.96E-06 | Activator of angiogenesis | Zheng *et al*., 2003[51] |
| *Enpep* | Glutamyl aminopeptidase | 2.42 / 4.98 | 2.04E-04 / 1.75E-06 | Marker of capillary EC | Li *et al*., 1993[52] |
| *Ephb4* | Eph receptor B4 | 3.10 / 3.37 | 1.17E-05 / 3.84E-06 | Increases vessel sprouting, mediates migration and proliferation of EC | Adams *et al*., 2001[53], Surawska *et al*., 2004[54] |
| *Hoxa3* | Homeo box A3 | 5.16 / 4.08 | 1.48E-06 / 1.96E-06 | Maintains EC program | Iacovino *et al*., 2010[55] |
| *Mrc2* | Mannose receptor C, type 2 | 4.67 / 3.72 | 7.11E-06 / 3.84E-06 | Expressed in EC | Engelholm *et al*., 2001[56] |
| *Myof* | Myoferlin | 5.22 / 6.14 | 1.17E-05 / 3.84E-06 | Stabilizes VEGFR2 in EC membrane | Bernatchez *et al*., 2007[57] |
| *Nab2* | NGFI-A binding protein 2 (EGR1 binding protein 2) | 4.09 / 2.69 | 1.48E-06 / 8.20E-06 | Inhibits vessel sprouting | Lucerna *et al*., 2002[58] |
| *Nid1* | Nidogen 1 | - / 5.98 | -/ 2.74E-06 | Prevents vessel sprouting | Semkova *et al*., 2014[59] |
| *Nos3* | Nitric oxide synthase 3, (endothelial cell) | 3.12 / 3.22 | 6.73E-06 / 2.77E-06 | Specifically expressed by EC, involved in migration | Aicher *et al.*, 2003[60], North *et al*., 2009[61], Noiri *et al*.,, 1997 |
| *Olfml3* | Olfactomedin-like 3 | 5.44 / 3.37 | 1.20E-06 / 5.90E-06 | Promotes angiogenesis | Miljkovic-Licina *et al.*, 2012[62] |
| *Plat* | Plasminogen activator, tissue | 4.22 / 14.91 | 1.17E-05 / 2.24E-07 | Promotes proliferation, migration and angiogenesis | Lin and Chuang, 2012[63] |
| *Pltp* | Phospholipid Transfer Protein | 4.30 / 5.44 | 9.64E-07 / 3.83E-07 | Expressed in brain EC | Manavalan *et al.*, 2014[64] |
| *Plvap* | Plasmalemma vesicle associated protein | 3.55 / 4.98 | 6.73E-06 / 1.10E-06 | Expressed in EC | Wisniewska-Kruk *et al*., 2012[65] |
| *Ptn* | Pleiotrophin | 8.55 / 16.02 | 1.20E-06 / 3.08E-07 | Induces vessel sprouting | Besse *et al.*, 2013[66] |
| *Ramp2* | Receptor (calcitonin) activity modifying protein 2 | 3.40 / 3.65 | 6.73E-06 / 2.74E-06 | Expressed in EC, pro-angiogenic | Guidolin, 2010[67], Fernandez-Sauze *et al.*, 2004[68] |
| *Ramp3* | Similar to receptor activity modifying protein 3 | 4.09 / 6.35 | 1.20E-06 / 2.24E-07 | Expressed in EC, pro-angiogenic | Guidolin, 2010[67], Fernandez-Sauze *et al.*, 2004[68] |
| *S1pr1* | Sphingosine-1 phosphate receptor 1 | 4.05 / 3.77 | 1.96E-06 / 1.75E-06 | Restricts vessel sprouting | Gaengel *et al.*, 2012[69] |
| *Sema3f* | Semaphorin 3F | 4.08 / 3.66 | 1.96E-06 / 4.65E-06 | Inhibits tumor angiogenesis | Guttman-Raviv *et al.*, 2007[70], Guo *et al.*, 2013[71] |
| *Slco2a1* | Solute carrier organic anion transporter family, member 2A1 | 3.16 / 4.34 | 6.73E-06 / 2.68E-06 | Expressed in EC | Topper *et al.*, 1998[72] |
| *Smad6* | SMAD family member 6 | 3.91 / 3.74 | 1.96E-06 / 1.75E-06 | Expressed in EC | Sandusky, 2002[73] |
| *Sox18* | SRY-box containing gene 18 | 3.17 / 3.44 | 2.21E-05 / 6.87E-06 | Promotes angiogenesis | Samant *et al.*, 2011[74] |
| *Tek* | Endothelial-specific receptor tyrosine kinase | 3.48 / 4.57 | 6.73E-06 / 1.10E-06 | Promotes angiogenesis | Martin *et al.*, 2008[75], Li *et al*., 2009[76] |
| *Tgfbr2* | Transforming growth factor, beta receptor II | 3.18 / 2.98 | 1.30E-05 / 8.20E-06 | Maintaining vascular integrity | Allinson *et al.*, 2012[77] |
| *Wwtr1* | WW domain containing transcription regulator 1 | 3.88 / 4.21 | 5.81E-06 / 1.98E-06 | Involved in promoting survival of EC | Dupont *et al.*, 2011[78] |
| **Mesenchymal stem cells marker,** **recruitment and/or function** | | | | | |
| *Anpep* | Alanyl (membrane) aminopeptidase | 3.59 / 4.75 | 1.48E-06 / 6.32E-07 | Marker of MSC | Jones *et al*., 2010[79] |
| *Bmp1* | Bone morphogenetic protein 1 | 9.11 / 4.29 | 1.34E-06 / 3.84E-06 | Enhances differentiation of MSCs towards osteoblasts | Grgurevic *et al.*, 2011[3] |
| *Ccdc80* | Coiled-coil domain containing 80 | 4.28 / 3.97 | 2.19E-05 / 7.54E-06 | Suggested stemness marker for BMSC | Liu *et al.*, 2004[80] |
| *Cd109* | Cd109 antigen | 12.62 / 4.84 | 1.20E-06 / 8.20E-06 | MSC surface marker | Giesert *et al.*, 2003[81] |
| *Cd200* | Cd200 antigen | 4.41 / 2.83 | 1.20E-06 / 5.90E-06 | MSC surface marker; MSCs that express high levels of Cd200 have increased osteogenic potential | Rostovskaya and Anastassiadis, 2012[82] |
| *Cxcl16* | Chemokine (C-X-C motif) ligand 16 | 4.44 / 8.62 | 1.02E-04 / 4.97E-06 | Recruits MSCs | Smith *et al.*, 2012[48] |
| *Enpep* | Glutamyl aminopeptidase | 2.42 / 4.98 | 2.04E-04 / 1.75E-06 | Marker of MSC | Chan *et al.*, 2013[8] |
| *Ltbp3* | Latent transforming growth factor beta binding protein 3 | 3.19 / 2.47 | 1.48E-06 / 3.88E-06 | Expressed by MSCs | Koli *et al.*, 2008[12] |
| *Pdlim4* | PDZ and LIM domain 4 | 4.12 / 4.80 | 5.09E-06 / 1.75E-06 | Expressed in bone marrow stromal cells | Bashirova *et al*., 1998[83] |
| *Plvap* | Plasmalemma vesicle associated protein | 3.55 / 4.98 | 6.73E-06 / 1.10E-06 | Maintenance of stemness properties | Basciano *et al*., 2011[84] |
| *S1pr1* | Sphingosine-1 phosphate receptor 1 | 4.05 / 3.77 | 1.96E-06 / 1.75E-06 | Expressed on MSCs; chemokine S1P stimulates mesenchymal cell chemotaxis | Quint *et al.*, 2013[85] |
| *Sp7* | Sp7 transcription factor 7 | 6.17 / 1.36 | 1.20E-06 / 6.17E-02 | Expressed in mesenchymal progenitor cells | Kaback *et al.*, 2008[86] |
| *Wwtr1* | WW domain containing transcription regulator 1 | 3.88 / 4.21 | 5.81E-06 / 1.98E-06 | Expressed in MSCs | Dupont *et al.*, 2011[78] |
| **Hematopoietic stem cell niche components** | | | | | |
| *Abca1* | ATP-binding cassette, sub-family A (ABC1), member 1 | 3.32 / 4.05 | 6.73E-06 / 4.65E-06 | Regulation of HSC niche | Westerterp *et al.*, 2012[87] |
| *Cdh2* | Cadherin 2 | 5.94 / 1.86 | 1.68E-05 / 1.81E-02 | Mediates homophilic adhesion to osteoblasts in the HSC niche | Zhang *et al*., 2003[88], Arai *et al*., 2102[89] |
| *Epha3* | Eph receptor A3 | 17.53 / 14.39 | 1.20E-06 / 1.51E-06 | Homing factor for stem cells to the bone marrow | Ting *et al.*, 2010[90] |
| *Lamb1* | Laminin B1 subunit 1 | 4.38 / 5.72 | 2.21E-05 / 3.93E-06 | Laminins facilitate survival and self-renewal of pluripotent stem cells | Gu *et al*, 2003[91], Rodin *et al.*, 2010[92] |
| *Nos3* | Nitric oxide synthase 3, (endothelial cell) | 3.12 / 3.22 | 6.73E-06 / 2.77E-06 | Regulates maintenance and mobilization of stem cells in the bone marrow | Aicher *et al.*, 2003[60], North *et al*., 2009[61] |
| *Olfml3* | Olfactomedin-like 3 | 5.44 / 3.37 | 1.20E-06 / 5.90E-06 | Regulates assembly of HSC perivascular niche | Miljkovic-Licina *et al.*, 2012[62] |
| *Ptn* | Pleiotrophin | 8.55 / 16.02 | 1.20E-06 / 3.08E-07 | Regulates the maintenance of the HSC pool; homing factor for stem cells to the bone marrow | Himburg *et al.*, 2012[93] |
| *S1pr1* | Sphingosine-1 phosphate receptor 1 | 4.05 / 3.77 | 1.96E-06 / 1.75E-06 | Expressed on HSCs; S1P (=ligand) facilitates the egress of committed hematopoietic progenitors from the BM into the blood | Juarez *et al.*, 2012[94] |
| *Sstr2* | Somatostatin receptor 2 | 8.70 / 1.98 | 1.59E-07 / 1.45E-04 | Expressed on HSCs, involved in bone marrow homing | Oomen *et al.*, 2002[95] |
| *Tek* | Endothelial-specific receptor tyrosine kinase | 3.48 / 4.57 | 6.73E-06 / 1.10E-06 | Expressed in HSCs; maintains quiescent status of HSCs | Yano *et al.*, 1997[96], Martin *et al.*, 2008[75] |
| **Cancer cell niche components** | | | | | |
| *Bmp1* | Bone morphogenetic protein 1 | 9.11 / 4.29 | 1.34E-06 / 3.84E-06 | Promotes proteolytic activation of lysyl oxidase | Maruhashi *et al.*, 2010[97], Erler *et al*., 2009[98] |
| *Epha3* | Eph receptor A3 | 17.53 / 14.39 | 1.20E-06 / 1.51E-06 | Promotes angiogenesis, expressed on tumor-initiating cell population, maintains tumor cells in a less differentiated state | Xi *et al.*, 2012[99], Day *et al.*, 2013[100] |
| *Ephb4* | Eph receptor B4 | 3.10 / 3.37 | 1.17E-05 / 3.84E-06 | Deregulated Ephb4-ephrinb2 signaling may contribute to the acquisition of a metastatic phenotype; modulates angio- / lymph-angiogenesis | Kaenel *et al.*, 2011[101], Abéngozar *et al.*, 2012[102] |
| *Lamb1* | Laminin B1, subunit 1 | 4.38 / 5.72 | 2.21E-05 / 3.93E-06 | Displays anti-adhesive functions and has potential implications for cell migration during matrix remodeling; angiogenesis | Santos-Valle *et al.*, 2012[103], Patarroyo *et al.*, 2002[104], Ghajar *et al*., 2013[105] |
| *Ltbp4* | Latent transforming growth factor beta binding protein 4 | 3.37 / 7.37 | 6.73E-06 / 3.08E-07 | Modulates activation of latent TGFbeta | Ghajar *et al.,* 2013[105] |
| *Nid1* | Nidogen 1 | - / 5.98 | -/ 2.74E-06 | Overexpressed in BM-like microvascular niche *in vitro* | Ghajar *et al.,* 2013[105] |
| *Olfml3* | Olfactomedin-like 3 | 5.44 / 3.37 | 1.20E-06 / 5.90E-06 | Promotes angiogenesis and tumor growth | Miljkovic-Licina *et al.*, 2012[62] |
| *Ptn* | Pleiotrophin | 8.55 / 16.02 | 1.20E-06 / 3.08E-07 | Stimulates angiogenesis; CAFs; stimulates cancer cell growth | Perez-Pinera *et al.*, 2007[106], Diamantopoulou *et al.*, 2012 [107] |
| *S1pr1* | Sphingosine-1 phosphate receptor 1 | 4.05 / 3.77 | 1.96E-06 / 1.75E-06 | Regulates pre-metastatic niche; angiogenesis | Deng *et al.*, 2012[108], Yang *et al.*, 2013[109] |

Note: 96 genes corresponding to all genes more than 3 fold induced in both xenografts and the top 30 of VCaP and C4-2B xenografts were reviewed. Genes can be assigned to more than one category.

Abbreviations: BMSC, bone marrow stromal cell; CAF, cancer-associated fibroblast; EC, endothelial cells; ECM, extracellular matrix; FDR, false discovery rate; HSC, hematopoietic stem cell; MSC, mesenchymal stem cell; OPG, osteoprotegerin; PTH, parathyroid hormone; RANKL, receptor activator of NF-kappa-B ligand.

**References**

1. Kleyer A, Scholtysek C, Bottesch E, Hillienhof U, Beyer C, et al. (2012) Liver X receptors orchestrate osteoblast/osteoclast crosstalk and counteract pathologic bone loss. Journal of Bone and Mineral Research 27: 2442–2451. doi:10.1002/jbmr.1702.

2. Narisawa S, Yadav MC, Millán JL (2013) In vivo overexpression of tissue-nonspecific alkaline phosphatase increases skeletal mineralization and affects the phosphorylation status of osteopontin. Journal of Bone and Mineral Research. Available: http://eutils.ncbi.nlm.nih.gov/entrez/eutils/elink.fcgi?dbfrom=pubmed&id=23427088&retmode=ref&cmd=prlinks.

3. Grgurevic L, Macek B, Mercep M, Jelic M, Smoljanovic T, et al. (2011) Bone morphogenetic protein (BMP)1-3 enhances bone repair. Biochem Biophys Res Commun 408: 25–31. Available: http://eutils.ncbi.nlm.nih.gov/entrez/eutils/elink.fcgi?dbfrom=pubmed&id=21453682&retmode=ref&cmd=prlinks.

4. Lee L, Liu J, Manuel J, Gorczynski RM (2006) A role for the immunomodulatory molecules CD200 and CD200R in regulating bone formation. Immunology Letters 105: 150–158. doi:10.1016/j.imlet.2006.02.002.

5. Ferrari SL, Traianedes K, Thorne M, Lafage-Proust MH, Genever P, et al. (2000) A role for N-cadherin in the development of the differentiated osteoblastic phenotype. J Bone Miner Res 15: 198–208. doi:10.1359/jbmr.2000.15.2.198.

6. Hecht J, Seitz V, Urban M, Wagner F, Robinson PN, et al. (2007) Detection of novel skeletogenesis target genes by comprehensive analysis of a Runx2(-/-) mouse model. Gene Expr Patterns 7: 102–112. doi:10.1016/j.modgep.2006.05.014.

7. Murakami T, Saito A, Hino S-I, Kondo S, Kanemoto S, et al. (2009) Signalling mediated by the endoplasmic reticulum stress transducer OASIS is involved in bone formation. 11: 1205–1211. Available: http://eutils.ncbi.nlm.nih.gov/entrez/eutils/elink.fcgi?dbfrom=pubmed&id=19767743&retmode=ref&cmd=prlinks.

8. Chan CKF, Lindau P, Jiang W, Chen JY, Zhang LF, et al. (2013) Clonal precursor of bone, cartilage, and hematopoietic niche stromal cells. Proc Natl Acad Sci USA 110: 12643–12648. doi:10.1073/pnas.1310212110.

9. Martin TJ, Allan EH, Ho PWM, Gooi JH, Quinn JMW, et al. (2009) Communication Between EphrinB2 and EphB4 Within the Osteoblast Lineage. Advances in Experimental Medicine and Biology. Advances in Experimental Medicine and Biology. Boston, MA: Osteoimmunology, Vol. 658. pp. 51–60. doi:10.1007/978-1-4419-1050-9_6.

10. Kovacs CS, Chafe LL, Fudge NJ, Friel JK, Manley NR (2001) PTH regulates fetal blood calcium and skeletal mineralization independently of PTHrP. Endocrinology 142: 4983–4993. Available: http://eutils.ncbi.nlm.nih.gov/entrez/eutils/elink.fcgi?dbfrom=pubmed&id=11606467&retmode=ref&cmd=prlinks.

11. Tuckermann JP, Pittois K, Partridge NC, Merregaert J, Angel P (2000) Collagenase-3 (MMP-13) and integral membrane protein 2a (Itm2a) are marker genes of chondrogenic/osteoblastic cells in bone formation: sequential temporal, and spatial expression of Itm2a, alkaline phosphatase, MMP-13, and osteocalcin in the mouse. J Bone Miner Res 15: 1257–1265. doi:10.1359/jbmr.2000.15.7.1257.

12. Koli K, Ryynänen MJ, Keski-Oja J (2008) Latent TGF-beta binding proteins (LTBPs)-1 and -3 coordinate proliferation and osteogenic differentiation of human mesenchymal stem cells. 43: 679–688. Available: http://eutils.ncbi.nlm.nih.gov/entrez/eutils/elink.fcgi?dbfrom=pubmed&id=18672106&retmode=ref&cmd=prlinks.

13. Mukherjee A, Larson EA, Carlos AS, Belknap JK, Rotwein P, et al. (2012) Congenic mice provide in vivo evidence for a genetic locus that modulates intrinsic transforming growth factor β1-mediated signaling and bone acquisition. 27: 1345–1356. Available: http://eutils.ncbi.nlm.nih.gov/entrez/eutils/elink.fcgi?dbfrom=pubmed&id=22407846&retmode=ref&cmd=prlinks.

14. Clancy, Johnson, Rezvankhah, Wong, Resmini, et al. (2003) A gene expression profile for endochondral bone formation: oligonucleotide microarrays establish novel connections between known genes and BMP-2-induced bone formation in mouse quadriceps. 33: 18–18. Available: http://eutils.ncbi.nlm.nih.gov/entrez/eutils/elink.fcgi?dbfrom=pubmed&id=12919699&retmode=ref&cmd=prlinks.

15. Caley MP, Kogianni G, Adamarek A, Gronau JH, Rodriguez-Teja M, et al. (2011) TGFβ1-Endo180-dependent collagen deposition is dysregulated at the tumour-stromal interface in bone metastasis. J Pathol 226: 775–783. doi:10.1002/path.3958.

16. Chandra A, Lan S, Zhu J, Siclari VA, Qin L (2013) Epidermal Growth Factor Receptor (EGFR) Signaling Promotes Proliferation and Survival in Osteoprogenitors by Increasing Early Growth Response 2 (EGR2) Expression. Journal of Biological Chemistry 288: 20488–20498. doi:10.1074/jbc.M112.447250.

17. Aguirre J, Buttery L, O'Shaughnessy M, Afzal F, Fernandez de Marticorena I, et al. (2001) Endothelial nitric oxide synthase gene-deficient mice demonstrate marked retardation in postnatal bone formation, reduced bone volume, and defects in osteoblast maturation and activity. 158: 247–257. Available: http://eutils.ncbi.nlm.nih.gov/entrez/eutils/elink.fcgi?dbfrom=pubmed&id=11141498&retmode=ref&cmd=prlinks.

18. Tarroni P, Villa I, Mrak E, Zolezzi F, Mattioli M, et al. (2012) Microarray analysis of 1,25(OH)₂D₃ regulated gene expression in human primary osteoblasts. 113: 640–649. Available: http://eutils.ncbi.nlm.nih.gov/entrez/eutils/elink.fcgi?dbfrom=pubmed&id=21956231&retmode=ref&cmd=prlinks.

19. Bustos-Valenzuela JC, Fujita A, Halcsik E, Granjeiro JM, Sogayar MC (2011) Unveiling novel genes upregulated by both rhBMP2 and rhBMP7 during early osteoblastic transdifferentiation of C2C12 cells. 4: 370. Available: http://eutils.ncbi.nlm.nih.gov/entrez/eutils/elink.fcgi?dbfrom=pubmed&id=21943021&retmode=ref&cmd=prlinks.

20. Scheideler M, Elabd C, Zaragosi L-E, Chiellini C, Hackl H, et al. (2008) Comparative transcriptomics of human multipotent stem cells during adipogenesis and osteoblastogenesis. BMC Genomics 9: 340. doi:10.1186/1471-2164-9-340.

21. Sato YY, Takita HH, Ohata NN, Tamura MM, Kuboki YY (2002) Pleiotrophin regulates bone morphogenetic protein (BMP)-induced ectopic osteogenesis. 131: 877–886. Available: http://eutils.ncbi.nlm.nih.gov/entrez/eutils/elink.fcgi?dbfrom=pubmed&id=12038985&retmode=ref&cmd=prlinks.

22. Naot D, Callon KE, Grey A, Cooper GJ, Reid IR, et al. (2001) A potential role for adrenomedullin as a local regulator of bone growth. Endocrinology 142: 1849–1857. doi:10.1210/endo.142.5.8152.

23. Uzan B, de Vernejoul M-C, Cressent M (2004) RAMPs and CRLR expressions in osteoblastic cells after dexamethasone treatment. Biochem Biophys Res Commun 321: 802–808. doi:10.1016/j.bbrc.2004.07.037.

24. Phelps E, Bezouglaia O, Tetradis S, Nervina JM (2005) Parathyroid hormone induces receptor activity modifying protein-3 (RAMP3) expression primarily via 3“,5-”cyclic adenosine monophosphate signaling in osteoblasts. 77: 96–103. Available: http://eutils.ncbi.nlm.nih.gov/entrez/eutils/elink.fcgi?dbfrom=pubmed&id=16075364&retmode=ref&cmd=prlinks.

25. Fukada T, Civic N, Furuichi T, Shimoda S, Mishima K, et al. (2008) The zinc transporter SLC39A13/ZIP13 is required for connective tissue development; its involvement in BMP/TGF-beta signaling pathways. 3: e3642. Available: http://eutils.ncbi.nlm.nih.gov/entrez/eutils/elink.fcgi?dbfrom=pubmed&id=18985159&retmode=ref&cmd=prlinks.

26. Zhang Z, Xia W, He J, Zhang Z, Ke Y, et al. (2012) REPOR TExome Sequencing Identifies SLCO2A1 Mutationsas a Cause of Primary Hypertrophic Osteoarthropathy. The American Journal of Human Genetics 90: 125–132. doi:10.1016/j.ajhg.2011.11.019.

27. Seifert W, Kühnisch J, Tüysüz B, Specker C, Brouwers A, et al. (2012) Mutations in the prostaglandin transporter encoding gene SLCO2A1Cause primary hypertrophic osteoarthropathy and isolated digital clubbing. Hum Mutat 33: 660–664. doi:10.1002/humu.22042.

28. Chae Y-M, Heo S-H, Kim J-Y, Lee J-M, Ryoo H-M, et al. (2009) Upregulation of smpd3 via BMP2 stimulation and Runx2. 42: 86–90. Available: http://eutils.ncbi.nlm.nih.gov/entrez/eutils/elink.fcgi?dbfrom=pubmed&id=19250608&retmode=ref&cmd=prlinks.

29. Gao Y, Jheon A, Nourkeyhani H, Kobayashi H, Ganss B (2004) Molecular cloning, structure, expression, and chromosomal localization of the human Osterix (SP7) gene. Gene 341: 101–110. doi:10.1016/j.gene.2004.05.026.

30. Seo H-S, Serra R (2009) Tgfbr2 is required for development of the skull vault. 334: 481–490. Available: http://eutils.ncbi.nlm.nih.gov/entrez/eutils/elink.fcgi?dbfrom=pubmed&id=19699732&retmode=ref&cmd=prlinks.

31. Mizuhashi K, Kanamoto T, Ito M, Moriishi T, Muranishi Y, et al. (2012) OBIF, an osteoblast induction factor, plays an essential role in bone formation in association with osteoblastogenesis. Dev Growth Differ 54: 474–480. doi:10.1111/j.1440-169X.2012.01333.x.

32. Byun MR, Jeong H, Bae SJ, Kim AR, Hwang ES, et al. (2012) TAZ is required for the osteogenic and anti-adipogenic activities of kaempferol. 50: 364–372. Available: http://eutils.ncbi.nlm.nih.gov/entrez/eutils/elink.fcgi?dbfrom=pubmed&id=22108137&retmode=ref&cmd=prlinks.

33. Wang Y, Inger M, Jiang H, Tenenbaum H, Glogauer M (2013) CD109 Plays a Role in Osteoclastogenesis. PLoS ONE 8: e61213. doi:10.1371/journal.pone.0061213.

34. Cui W, Cuartas E, Ke J, Zhang Q, Einarsson HB, et al. (2007) CD200 and its receptor, CD200R, modulate bone mass via the differentiation of osteoclasts. 104: 14436–14441. Available: http://eutils.ncbi.nlm.nih.gov/entrez/eutils/elink.fcgi?dbfrom=pubmed&id=17726108&retmode=ref&cmd=prlinks.

35. Varin A, Pontikoglou C, Labat E, Deschaseaux F, Sensebé L (2013) CD200R/CD200 Inhibits Osteoclastogenesis: New Mechanism of Osteoclast Control by Mesenchymal Stem Cells in Human. PLoS ONE 8: e72831. doi:10.1371/journal.pone.0072831.s003.

36. Kim H-J, Hong JM, Yoon K-A, Kim N, Cho D-W, et al. (2012) Early growth response 2 negatively modulates osteoclast differentiation through upregulation of Id helix-loop-helix proteins. 51: 643–650. Available: http://eutils.ncbi.nlm.nih.gov/entrez/eutils/elink.fcgi?dbfrom=pubmed&id=22842221&retmode=ref&cmd=prlinks.

37. Xing L (2012) Osteoclast fusion and regulation by RANKL-dependent and independent factors. WJO 3: 212. doi:10.5312/wjo.v3.i12.212.

38. Yang JN, Allan EH, Anderson GI, Martin TJ, Minkin C (1997) Plasminogen activator system in osteoclasts. J Bone Miner Res 12: 761–768. doi:10.1359/jbmr.1997.12.5.761.

39. Coudert AE, Del Fattore A, Baulard C, Olaso R, Schiltz C, et al. (2014) Differentially expressed genes in autosomal dominant osteopetrosis type II osteoclasts reveal known and novel pathways for osteoclast biology. Lab Invest 94: 275–285. doi:10.1038/labinvest.2013.140.

40. Vaisman BL, Demosky SJ, Stonik JA, Ghias M, Knapper CL, et al. (2012) Endothelial expression of human ABCA1 in mice increases plasma HDL cholesterol and reduces diet-induced atherosclerosis. 53: 158–167. Available: http://eutils.ncbi.nlm.nih.gov/entrez/eutils/elink.fcgi?dbfrom=pubmed&id=22039582&retmode=ref&cmd=prlinks.

41. Ge G, Fernández CA, Moses MA, Greenspan DS (2007) Bone morphogenetic protein 1 processes prolactin to a 17-kDa antiangiogenic factor. Proc Natl Acad Sci U S A 104: 10010–10015. doi:10.1073/pnas.0704179104.

42. Gonzalez EM, Reed CC, Bix G, Fu J, Zhang Y, et al. (2005) BMP-1/Tolloid-like Metalloproteases Process Endorepellin, the Angiostatic C-terminal Fragment of Perlecan. Journal of Biological Chemistry 280: 7080–7087. doi:10.1074/jbc.M409841200.

43. Sowa G (2012) Caveolae, caveolins, cavins, and endothelial cell function: new insights: 1–13. doi:10.3389/fphys.2011.00120/abstract.

44. (null), (null), (null), (null), (null), et al. (2013) Human Platelet Antigen Genotyping and Expression of CD109 (Human Platelet Antigen 15) mRNA in Various Human Cell Types. BioMed Research International 2013: 1–5. Available: http://eutils.ncbi.nlm.nih.gov/entrez/eutils/elink.fcgi?dbfrom=pubmed&id=23509816&retmode=ref&cmd=prlinks.

45. Ko Y-C, Chien H-F, Jiang-Shieh Y-F, Chang C-Y, Pai M-H, et al. (2009) Endothelial CD200 is heterogeneously distributed, regulated and involved in immune cell-endothelium interactions. Journal of Anatomy 214: 183–195. Available: http://eutils.ncbi.nlm.nih.gov/entrez/eutils/elink.fcgi?dbfrom=pubmed&id=19166481&retmode=ref&cmd=prlinks.

46. Ohtani K, Suzuki Y, Eda S, Kawai T, Kase T, et al. (2001) The membrane-type collectin CL-P1 is a scavenger receptor on vascular endothelial cells. 276: 44222–44228. Available: http://eutils.ncbi.nlm.nih.gov/entrez/eutils/elink.fcgi?dbfrom=pubmed&id=11564734&retmode=ref&cmd=prlinks.

47. Miyagi H, Kanemoto S, Saito A, Asada R, Iwamoto H, et al. (2013) Transcriptional regulation of VEGFA by the endoplasmic reticulum stress transducer OASIS in ARPE-19 cells. PLoS ONE 8: e55155. Available: http://eutils.ncbi.nlm.nih.gov/entrez/eutils/elink.fcgi?dbfrom=pubmed&id=23383089&retmode=ref&cmd=prlinks.

48. Smith H, Whittall C, Weksler B, Middleton J (2012) Chemokines stimulate bidirectional migration of human mesenchymal stem cells across bone marrow endothelial cells. Stem cells and development 21: 476–486. Available: http://eutils.ncbi.nlm.nih.gov/entrez/eutils/elink.fcgi?dbfrom=pubmed&id=21513440&retmode=ref&cmd=prlinks.

49. Zorrilla LM, Sriperumbudur R, Gadsby JE (2010) Endothelin-1, endothelin converting enzyme-1 and endothelin receptors in the porcine corpus luteum. Domestic Animal Endocrinology 38: 75–85. Available: http://eutils.ncbi.nlm.nih.gov/entrez/eutils/elink.fcgi?dbfrom=pubmed&id=19783117&retmode=ref&cmd=prlinks.

50. López-Ongil S, Díez-Marqués M-L, Griera M, Rodríguez-Puyol M, Rodríguez-Puyol D (2005) Crosstalk between mesangial and endothelial cells: angiotensin II down-regulates endothelin-converting enzyme 1. Cell Physiol Biochem 15: 135–144. doi:10.1159/000083646.

51. Zheng H, Wasylyk C, Ayadi A, Abecassis J, Schalken JA, et al. (2003) The transcription factor Net regulates the angiogenic switch. 17: 2283–2297. Available: http://eutils.ncbi.nlm.nih.gov/entrez/eutils/elink.fcgi?dbfrom=pubmed&id=12975317&retmode=ref&cmd=prlinks.

52. Li L, Wang J, Cooper MD (1993) cDNA cloning and expression of human glutamyl aminopeptidase (aminopeptidase A). Genomics 17: 657–664. Available: http://eutils.ncbi.nlm.nih.gov/entrez/eutils/elink.fcgi?dbfrom=pubmed&id=8244382&retmode=ref&cmd=prlinks.

53. Adams RH, Diella F, Hennig S, Helmbacher F, Deutsch U, et al. (2001) The cytoplasmic domain of the ligand ephrinB2 is required for vascular morphogenesis but not cranial neural crest migration. Cell 104: 57–69. Available: http://eutils.ncbi.nlm.nih.gov/entrez/eutils/elink.fcgi?dbfrom=pubmed&id=11163240&retmode=ref&cmd=prlinks.

54. Surawska H, Ma PC, Salgia R (2004) The role of ephrins and Eph receptors in cancer. Cytokine Growth Factor Rev 15: 419–433. Available: http://eutils.ncbi.nlm.nih.gov/entrez/eutils/elink.fcgi?dbfrom=pubmed&id=15561600&retmode=ref&cmd=prlinks.

55. Iacovino M, Chong D, Szatmari I, Hartweck L, Rux D, et al. (2010) HoxA3 is an apical regulator of haemogenic endothelium. Nat Cell Biol 13: 72–78. Available: http://eutils.ncbi.nlm.nih.gov/entrez/eutils/elink.fcgi?dbfrom=pubmed&id=21170035&retmode=ref&cmd=prlinks.

56. Engelholm L (2001) The Urokinase Receptor Associated Protein (uPARAP/Endo180) A Novel Internalization Receptor Connected to the Plasminogen Activation System. Trends Cardiovasc Med 11: 7–13. Available: http://eutils.ncbi.nlm.nih.gov/entrez/eutils/elink.fcgi?dbfrom=pubmed&id=11413046&retmode=ref&cmd=prlinks.

57. Bernatchez PN, Acevedo L, Fernandez-Hernando C, Murata T, Chalouni C, et al. (2007) Myoferlin regulates vascular endothelial growth factor receptor-2 stability and function. 282: 30745–30753. Available: http://eutils.ncbi.nlm.nih.gov/entrez/eutils/elink.fcgi?dbfrom=pubmed&id=17702744&retmode=ref&cmd=prlinks.

58. Lucerna M (2002) NAB2, a Corepressor of EGR-1, Inhibits Vascular Endothelial Growth Factor-mediated Gene Induction and Angiogenic Responses of Endothelial Cells. Journal of Biological Chemistry 278: 11433–11440. doi:10.1074/jbc.M204937200.

59. Semkova I, Kociok N, Karagiannis D, Nischt R, Smyth N, et al. (2014) Experimental Eye Research. Exp Eye Res 118: 80–88. doi:10.1016/j.exer.2013.11.006.

60. Aicher A, Heeschen C, Mildner-Rihm C, Urbich C, Ihling C, et al. (2003) Essential role of endothelial nitric oxide synthase for mobilization of stem and progenitor cells. Nat Med 9: 1370–1376. Available: http://eutils.ncbi.nlm.nih.gov/entrez/eutils/elink.fcgi?dbfrom=pubmed&id=14556003&retmode=ref&cmd=prlinks.

61. North TE, Goessling W, Peeters M, Li P, Ceol C, et al. (2009) Hematopoietic Stem Cell Development Is Dependent on Blood Flow. Cell 137: 736–748. doi:10.1016/j.cell.2009.04.023.

62. Miljkovic-Licina M, Hammel P, Garrido-Urbani S, Lee BP-L, Meguenani M, et al. (2012) Targeting olfactomedin-like 3 inhibits tumor growth by impairing angiogenesis and pericyte coverage. Molecular Cancer Therapeutics 11: 2588–2599. Available: http://eutils.ncbi.nlm.nih.gov/entrez/eutils/elink.fcgi?dbfrom=pubmed&id=23002094&retmode=ref&cmd=prlinks.

63. Lin Z-Y, Chuang W-L (2012) Genes responsible for the characteristics of primary cultured invasive phenotype hepatocellular carcinoma cells. Biomedicine & pharmacotherapy = Biomédecine & pharmacothérapie 66: 454–458. Available: http://eutils.ncbi.nlm.nih.gov/entrez/eutils/elink.fcgi?dbfrom=pubmed&id=22681909&retmode=ref&cmd=prlinks.

64. Manavalan APC, Kober A, Metso J, Lang I, Becker T, et al. (2014) Phospholipid transfer protein is expressed in cerebrovascular endothelial cells and involved in high density lipoprotein biogenesis and remodeling at the blood-brain barrier. Journal of Biological Chemistry 289: 4683–4698. Available: http://eutils.ncbi.nlm.nih.gov/entrez/eutils/elink.fcgi?dbfrom=pubmed&id=24369175&retmode=ref&cmd=prlinks.

65. Wisniewska-Kruk J, Hoeben KA, Vogels IMC, Gaillard PJ, Van Noorden CJF, et al. (2012) A novel co-culture model of the blood-retinal barrier based on primary retinal endothelial cells, pericytes and astrocytes. Exp Eye Res 96: 181–190. Available: http://eutils.ncbi.nlm.nih.gov/entrez/eutils/elink.fcgi?dbfrom=pubmed&id=22200486&retmode=ref&cmd=prlinks.

66. Besse S, Comte R, Fréchault S, Courty J, Joël de L, et al. (2013) Pleiotrophin promotes capillary-like sprouting from senescent aortic rings. Cytokine 62: 44–47. Available: http://eutils.ncbi.nlm.nih.gov/entrez/eutils/elink.fcgi?dbfrom=pubmed&id=23481101&retmode=ref&cmd=prlinks.

67. Guidolin (2010) Involvement of vascular endothelial growth factor signaling in CLR/RAMP1 and CLR/RAMP2-mediated pro-angiogenic effect of intermedin on human vascular endothelial cells. Int J Mol Med 26: 289–294. Available: http://eutils.ncbi.nlm.nih.gov/entrez/eutils/elink.fcgi?dbfrom=pubmed&id=20596610&retmode=ref&cmd=prlinks.

68. Fernandez-Sauze S, Delfino C, Mabrouk K, Dussert C, Chinot O, et al. (2004) Effects of adrenomedullin on endothelial cells in the multistep process of angiogenesis: Involvement of CRLR/RAMP2 and CRLR/RAMP3 receptors. Int J Cancer 108: 797–804. Available: http://eutils.ncbi.nlm.nih.gov/entrez/eutils/elink.fcgi?dbfrom=pubmed&id=14712479&retmode=ref&cmd=prlinks.

69. Gaengel K, Niaudet C, Hagikura K, Laviña B, Siemsen BL, et al. (2012) The sphingosine-1-phosphate receptor S1PR1 restricts sprouting angiogenesis by regulating the interplay between VE-cadherin and VEGFR2. Dev Cell 23: 587–599. Available: http://eutils.ncbi.nlm.nih.gov/entrez/eutils/elink.fcgi?dbfrom=pubmed&id=22975327&retmode=ref&cmd=prlinks.

70. Guttmann-Raviv N, Shraga-Heled N, Varshavsky A, Guimaraes-Sternberg C, Kessler O, et al. (2007) Semaphorin-3A and semaphorin-3F work together to repel endothelial cells and to inhibit their survival by induction of apoptosis. 282: 26294–26305. Available: http://eutils.ncbi.nlm.nih.gov/entrez/eutils/elink.fcgi?dbfrom=pubmed&id=17569671&retmode=ref&cmd=prlinks.

71. Guo H-F, Li X, Parker MW, Waltenberger J, Becker PM, et al. (2013) Mechanistic Basis for the Potent Anti-Angiogenic Activity of Semaphorin 3F. Biochemistry 52: 7551–7558. doi:10.1021/bi401034q.

72. Topper JN, Cai J, Stavrakis G, Anderson KR, Woolf EA, et al. (1998) Human Prostaglandin Transporter Gene (hPGT) is Regulated by Fluid Mechanical Stimuli in Cultured Endothelial Cells and Expressed in Vascular Endothelium in Vivo. Circulation 98: 2396–2403. Available: http://eutils.ncbi.nlm.nih.gov/entrez/eutils/elink.fcgi?dbfrom=pubmed&id=9832484&retmode=ref&cmd=prlinks.

73. Sandusky G (2002) Modulation of Thrombomodulin-dependent Activation of Human Protein C through Differential Expression of Endothelial Smads. Journal of Biological Chemistry 277: 49815–49819. Available: http://eutils.ncbi.nlm.nih.gov/entrez/eutils/elink.fcgi?dbfrom=pubmed&id=12407115&retmode=ref&cmd=prlinks.

74. Samant GV, Schupp MO, Francois M, Moleri S, Kothinti RK, et al. (2011) Sox factors transcriptionally regulate ROBO4 gene expression in developing vasculature in zebrafish. J Biol Chem 286: 30740–30747. Available: http://eutils.ncbi.nlm.nih.gov/entrez/eutils/elink.fcgi?dbfrom=pubmed&id=21730073&retmode=ref&cmd=prlinks.

75. Martin V, Liu D, Fueyo J, Gomez-Manzano C (2008) Tie2: a journey from normal angiogenesis to cancer and beyond. Histol Histopathol 23: 773–780. Available: http://eutils.ncbi.nlm.nih.gov/entrez/eutils/elink.fcgi?dbfrom=pubmed&id=18366015&retmode=ref&cmd=prlinks.

76. Li XM, Hu Z, Jorgenson ML, Slayton WB (2009) High Levels of Acetylated Low-Density Lipoprotein Uptake and Low Tyrosine Kinase With Immunoglobulin and Epidermal Growth Factor Homology Domains-2 (Tie2) Promoter Activity Distinguish Sinusoids From Other Vessel Types in Murine Bone Marrow. Circulation 120: 1910–1918. doi:10.1161/CIRCULATIONAHA.109.871574.

77. Allinson KR, Lee HS, Fruttiger M, McCarty JH, McCarty J, et al. (2012) Endothelial expression of TGFβ type II receptor is required to maintain vascular integrity during postnatal development of the central nervous system. PLoS ONE 7: e39336. Available: http://eutils.ncbi.nlm.nih.gov/entrez/eutils/elink.fcgi?dbfrom=pubmed&id=22745736&retmode=ref&cmd=prlinks.

78. Dupont S, Morsut L, Aragona M, Enzo E, Giulitti S, et al. (2011) Role of YAP/TAZ in mechanotransduction. Nature 474: 179–183. doi:10.1038/nature10137.

79. Jones E, English A, Churchman SM, Kouroupis D, Boxall SA, et al. (2010) Large-scale extraction and characterisation of CD271 +multipotential stromal cells (MSCs) from trabecular bone in health and osteoarthritis: Implications for bone regeneration strategies based on minimally-cultured MSCs. Arthritis & Rheumatism: NA–NA. doi:10.1002/art.27451.

80. Liu Y, Monticone M, Tonachini L, Mastrogiacomo M, Marigo V, et al. (2004) URB expression in human bone marrow stromal cells and during mouse development. 322: 497–507. Available: http://eutils.ncbi.nlm.nih.gov/entrez/eutils/elink.fcgi?dbfrom=pubmed&id=15325258&retmode=ref&cmd=prlinks.

81. Giesert C, Marxer A, Sutherland DR, Schuh AC, Kanz L, et al. (2003) Antibody W7C5 defines a CD109 epitope expressed on CD34+ and CD34- hematopoietic and mesenchymal stem cell subsets. Ann N Y Acad Sci 996: 227–230.

82. Rostovskaya M, Anastassiadis K (2012) Differential expression of surface markers in mouse bone marrow mesenchymal stromal cell subpopulations with distinct lineage commitment. PLoS ONE 7: e51221. Available: http://eutils.ncbi.nlm.nih.gov/entrez/eutils/elink.fcgi?dbfrom=pubmed&id=23236457&retmode=ref&cmd=prlinks.

83. Bashirova AA, Markelov ML, Shlykova TV, Levshenkova EV, Alibaeva RA, et al. (1998) The human RIL gene: mapping to human chromosome 5q31.1, genomic organization and alternative transcripts. Gene 210: 239–245.

84. Basciano L, Nemos C, Foliguet B, de Isla N, de Carvalho M, et al. (2011) Long term culture of mesenchymal stem cells inhypoxia promotes a genetic program maintainingtheir undifferentiated and multipotent status. BMC Cell Biology 12: 12. doi:10.1186/1471-2121-12-12.

85. Quint P, Ruan M, Pederson L, Kassem M, Westendorf JJ, et al. (2013) Sphingosine 1-phosphate (S1P) receptors 1 and 2 coordinately induce mesenchymal cell migration through S1P activation of complementary kinase pathways. J Biol Chem 288: 5398–5406. Available: http://eutils.ncbi.nlm.nih.gov/entrez/eutils/elink.fcgi?dbfrom=pubmed&id=23300082&retmode=ref&cmd=prlinks.

86. Kaback LA, Soung DY, Naik A, Smith N, Schwarz EM, et al. (2008) Osterix/Sp7 regulates mesenchymal stem cell mediated endochondral ossification. J Cell Physiol 214: 173–182. Available: http://eutils.ncbi.nlm.nih.gov/entrez/eutils/elink.fcgi?dbfrom=pubmed&id=17579353&retmode=ref&cmd=prlinks.

87. Westerterp M, Gourion-Arsiquaud S, Murphy AJ, Shih A, Cremers S, et al. (2012) Regulation of hematopoietic stem and progenitor cell mobilization by cholesterol efflux pathways. Cell Stem Cell 11: 195–206. Available: http://eutils.ncbi.nlm.nih.gov/entrez/eutils/elink.fcgi?dbfrom=pubmed&id=22862945&retmode=ref&cmd=prlinks.

88. Zhang J, Niu C, Ye L, Huang H, He X, et al. (2003) Identification of the haematopoietic stem cell niche and control of the niche size. Nature 425: 2837–2850. Available: http://www.jem.org/cgi/doi/10.1084/jem.20090778.

89. Arai F, Yoshihara H, Hosokawa K, Nakamura Y, Gomei Y, et al. (2009) Niche Regulation of Hematopoietic Stem Cells in the Endosteum. Ann N Y Acad Sci 1176: 36–46. Available: http://eutils.ncbi.nlm.nih.gov/entrez/eutils/elink.fcgi?dbfrom=pubmed&id=19796231&retmode=ref&cmd=prlinks.

90. Ting MJ, Day BW, Spanevello MD, Boyd AW (2010) Activation of ephrin A proteins influences hematopoietic stem cell adhesion and trafficking patterns. Exp Hematol 38: 1087–1098. doi:10.1016/j.exphem.2010.07.007.

91. Gu Y-C, Kortesmaa J, Tryggvason K, Persson J, Ekblom P, et al. (2003) Laminin isoform-specific promotion of adhesion and migration of human bone marrow progenitor cells. Blood 101: 877–885. doi:10.1182/blood-2002-03-0796.

92. Rodin S, Domogatskaya A, Ström S, Hansson EM, Chien KR, et al. (2010) long-term self-renewal of human pluripotent stem cells on human recombinant laminin-511. Nat Biotechnol 28: 611–615. doi:10.1038/nbt.1620.

93. Himburg HA, Harris JR, Ito T, Daher P, Russell JL, et al. (2012) Pleiotrophin regulates the retention and self-renewal of hematopoietic stem cells in the bone marrow vascular niche. Cell Rep 2: 964–975. doi:10.1016/j.celrep.2012.09.002.

94. Juarez JG, Harun N, Thien M, Welschinger R, Baraz R, et al. (2012) Sphingosine-1-phosphate facilitates trafficking of hematopoietic stem cells and their mobilization by CXCR4 antagonists in mice. Blood 119: 707–716. Available: http://eutils.ncbi.nlm.nih.gov/entrez/eutils/elink.fcgi?dbfrom=pubmed&id=22049516&retmode=ref&cmd=prlinks.

95. Oomen SPMA, van Hennik PB, Antonissen C, Lichtenauer-Kaligis EGR, Hofland LJ, et al. (2002) Somatostatin is a selective chemoattractant for primitive (CD34(+)) hematopoietic progenitor cells. 30: 116–125. Available: http://eutils.ncbi.nlm.nih.gov/entrez/eutils/elink.fcgi?dbfrom=pubmed&id=11823046&retmode=ref&cmd=prlinks.

96. Yano M, Iwama A, Nishio H, Suda J, Takada G, et al. (1997) Expression and function of murine receptor tyrosine kinases, TIE and TEK, in hematopoietic stem cells. Blood 89: 4317–4326. Available: http://eutils.ncbi.nlm.nih.gov/entrez/eutils/elink.fcgi?dbfrom=pubmed&id=9192754&retmode=ref&cmd=prlinks.

97. Maruhashi T, Kii I, Saito M, Kudo A (2010) Interaction between periostin and BMP-1 promotes proteolytic activation of lysyl oxidase. J Biol Chem 285: 13294–13303. doi:10.1074/jbc.M109.088864.

98. Erler JT, Bennewith KL, Cox TR, Lang G, Bird D, et al. (2009) Hypoxia-induced lysyl oxidase is a critical mediator of bone marrow cell recruitment to form the premetastatic niche. Cancer Cell 15: 35–44. doi:10.1016/j.ccr.2008.11.012.

99. Xi H-Q, Wu X-S, Wei B, Chen L (2012) Aberrant expression of EphA3 in gastric carcinoma: correlation with tumor angiogenesis and survival. J Gastroenterol 47: 785–794. Available: http://eutils.ncbi.nlm.nih.gov/entrez/eutils/elink.fcgi?dbfrom=pubmed&id=22350700&retmode=ref&cmd=prlinks.

100. Day BW, Stringer BW, Al-Ejeh F, Ting MJ, Wilson J, et al. (2013) EphA3 Maintains Tumorigenicity and Isa Therapeutic Target in Glioblastoma Multiforme. Cancer Cell 23: 238–248. doi:10.1016/j.ccr.2013.01.007.

101. Kaenel P, Schwab C, Mülchi K, Wotzkow C, Andres A-C (2011) Preponderance of cells with stem cell characteristics in metastasising mouse mammary tumours induced by deregulated EphB4 and ephrin-B2 expression. Int J Oncol 38: 151–160. Available: http://eutils.ncbi.nlm.nih.gov/entrez/eutils/elink.fcgi?dbfrom=pubmed&id=21109936&retmode=ref&cmd=prlinks.

102. Abéngozar MA, de Frutos S, Ferreiro S, Soriano J, Perez-Martinez M, et al. (2012) Blocking ephrinB2 with highly specific antibodies inhibits angiogenesis, lymphangiogenesis, and tumor growth. Blood 119: 4565–4576. Available: http://eutils.ncbi.nlm.nih.gov/entrez/eutils/elink.fcgi?dbfrom=pubmed&id=22446484&retmode=ref&cmd=prlinks.

103. Santos-Valle P, Guijarro-Muñoz I, Cuesta AM, Alonso-Camino V, Villate M, et al. (2012) The heterotrimeric laminin coiled-coil domain exerts anti-adhesive effects and induces a pro-invasive phenotype. PLoS ONE 7: e39097. Available: http://eutils.ncbi.nlm.nih.gov/entrez/eutils/elink.fcgi?dbfrom=pubmed&id=22723936&retmode=ref&cmd=prlinks.

104. Patarroyo M, Tryggvason K, Virtanen I (2002) Laminin isoforms in tumor invasion, angiogenesis and metastasis. Semin Cancer Biol 12: 197–207. Available: http://eutils.ncbi.nlm.nih.gov/entrez/eutils/elink.fcgi?dbfrom=pubmed&id=12083850&retmode=ref&cmd=prlinks.

105. Ghajar CM, Peinado H, Mori H, Matei IR, Evason KJ, et al. (2013) The perivascular niche regulates breast tumour dormancy. Nat Cell Biol 15: 807–817. doi:10.1371/journal.pone.0005798.

106. Perez-Pinera P, Chang Y, Deuel TF (2007) Pleiotrophin, a multifunctional tumor promoter through induction of tumor angiogenesis, remodeling of the tumor microenvironment, and activation of stromal fibroblasts. Cell Cycle 6: 2877–2883.

107. Diamantopoulou Z, Kitsou P, Menashi S, Courty J, Katsoris P (2012) Loss of Receptor Protein Tyrosine Phosphatase  /  (RPTP / ) Promotes Prostate Cancer Metastasis. Journal of Biological Chemistry 287: 40339–40349. doi:10.1074/jbc.M112.405852.

108. Deng J, Liu Y, Lee H, Herrmann A, Zhang W, et al. (2012) S1PR1-STAT3 signaling is crucial for myeloid cell colonization at future metastatic sites. Cancer Cell 21: 642–654. Available: http://eutils.ncbi.nlm.nih.gov/entrez/eutils/elink.fcgi?dbfrom=pubmed&id=22624714&retmode=ref&cmd=prlinks.

109. Yang L, Yang L, Yang L, Yang L, Yue S, et al. (2013) Sphingosine Kinase/Sphingosine 1-Phosphate (S1P)/S1P Receptor Axis is Involved in Liver Fibrosis-Associated Angiogenesis. Journal of hepatology. Available: http://eutils.ncbi.nlm.nih.gov/entrez/eutils/elink.fcgi?dbfrom=pubmed&id=23466305&retmode=ref&cmd=prlinks.
