# Supplementary material for: The Molecular Signature of the Stroma Response in Prostate Cancer-Induced Osteoblastic Bone Metastasis Highlights Expansion of Hematopoietic and Prostate Epithelial Stem Cell Niches
Source: PLoS One. 2014 Dec 8;9(12):e114530. doi: 10.1371/journal.pone.0114530 (PMC4259356; doi:10.1371/journal.pone.0114530)
Supplement: S1 File — This file contains an extended version of the Material and Methods, including references, one Figure and 11 Tables. (ZIP) [file pone.0114530.s010.zip › Supporting File/File S1.docx]

**Extended Materials and Methods**

**Xenografts**

Male CB17 mice with severe combined immunodeficiency (SCID) (Charles River, L’Arblesle, France) were 8 weeks old for intra-osseous inoculation of cancer cells and 6 weeks old for orthotopic and subcutaneous implantation.

For intra-osseous xenografts, osteoinductive PCa cells (C4-2B and VCaP; 5x10E05 cells), osteolytic PCa cells (PC-3; 2’500 cells) and immortalized, non-tumorigenic human prostate epithelial Ep156T cells (5x10E05 cells and 2’500 cells), in a total volume of 5μl, were inoculated in the bone marrow cavity of the left tibia [1]. The inoculation of Ep156T cells served as a control for a possible BM/B reaction to the local injection of human cells. As further controls, tibiae were collected from animals (sham) injected with phosphate-buffered saline (PBS) and from animals not subjected to surgery (intact). Development of bone lesions was followed by radiography at two weeks intervals for VCaP and C4-2B and at weekly intervals for PC-3 cells (MX-20, Faxitron X-Ray Corporation, Edimex, Le Plessis, France). Mice xenografted with VCaP and Ep156T cells were sacrificed after 6 weeks, those inoculated with C4-2B after 8 weeks. In order to correct for differences in growth kinetics and osteoinductive potential between VCaP and C4-2B, experimental endpoint was set based on radiographic evidence of osteoblastic reaction. PC-3 xenografts and their Ep156T controls animals were sacrificed after 33 days. Sham-operated and intact animals were sacrificed at all 3 time points. Tumor-bearing and contra-lateral tibiae of VCaP, C4-2B and PC-3 xenografted mice and left tibiae of Ep156T xenografted, sham and intact mice were excised and stored in RNAlater (Qiagen, Hombrechtikon, Switzerland) for subsequent RNA isolation. For histological studies tibiae were fixed in 4% paraformaldehyde, decalcified and embedded in paraffin.

VCaP cells were inoculated orthotopically (intra-prostate), while C4-2B*luc* and VCaP cells were implanted subcutaneously. For both types of xenografts, a suspension of 10E06 cells was mixed with collagen (Collagen Type I from rat tail, BD Biosciences, Allschwil, Switzerland). Prostates implanted with collagen pellets and dermal tissue served as control. Subcutaneous tumors were excised after 9 weeks and orthotopic tumors were excised after 10 weeks.

**Identification of cross-hybridizing probes**

Tissue RNA, which contained a mix of human and mouse sequences, was hybridized on Affymetrix GeneChip Mouse Genome 430A 2.0 arrays. To reduce the impact of cross-species hybridization on gene expression signals, we redefined probe sets in order to use only probes considered mouse-specific.

First, a computational mask was constructed by analyzing the sequence similarities of mouse probes with BLAST against the UniGene human database. Two exclusion criteria were defined: a stretch 20 or more matching nucleotides or 1 internal gap in a stretch of at least 21 matching nucleotides. This resulted in 52’909 (21.26 %) potentially cross-hybridizing cDNA-probes, which were excluded from the total 248’864 cDNA-probes on the mouse arrays.

Second, experimental cross-hybridization of cRNA from human cells (C4-2B and VCaP) on the mouse arrays was performed. A total number of 20’348 probes with background-adjusted log_2_ intensity above 9 were excluded. 10’366 probes were defined as “cross-hybridizing” by both the computational mask and experimental cross-hybridization. Only probe sets with at least 4 probes remaining after the filtering were used for further analysis. This was the case for 20,785 (185’973 cDNA-probes) out of 22,626 probe sets corresponding to a total of 13’217 (95.37%) genes, which were therefore considered mouse specific and used for the gene expression analysis.

Ultimately, 62’891 (25.27%) cDNA-probes, corresponding to 1’841 probe sets (8.14%), were excluded (Supporting File Table 1). This led to a total of 590 genes (Supporting File Table 1), which were not considered because none of their probe sets was retained. This list of excluded probe sets is universal and, thus, can be potentially applied to any other human cancer xenograft mouse model.

Probe set intensity was quantified with a modified version of the robust multi-array analysis (RMA) [2], in which we introduced our probe set exclusion step after the standard background subtraction and before quantile normalization and summarization by median polish.

**Quantitative RT-PCR**

All human and mouse specific gene expression assays are listed in Table 2.

**Immunofluorescence and immunohistochemistry**

Double immunofluorescent staining was performed with anti-smooth muscle actin and anti-periostin antibodies. DyLight 488 and 594-conjugated secondary antibodies (Jackson ImmunoResearch, Milan Analytica, Rheinfelden, Switzerland) were used for the detection.

All antibodies used for immunofluorescence and immunoperoxidase staining are listed in Table 3.

**Stroma specificity of the OB-BMST**

To verify the stroma specificity of the OB-BMST, we investigated whether pan-epithelial and/or prostate epithelial cell-specific markers were among the differentially expressed genes (Supporting File Table 4). This would be an indication of cross-reactivity with human RNA derived from PCa cells. Out of 26 pan-epithelial and/or prostate epithelial cell-specific marker genes, 25 were absent in the OB-BMST, the only exception being junction plakoglobin (*Jup*). However, this gene encodes a component of intercellular junctions, which is also expressed in endothelial cells (ECs) [3]. Furthermore, a literature review of 15 selected genes strongly up-regulated in the OB-BMST confirmed the stromal origin for all (Supporting File Table 5).

The differential expression and stroma specificity of selected genes was validated by RT-qPCR on RNA samples extracted from control and cancer cell-xenografted bones using mouse-specific probes. Seven highly up-regulated OB-BMST genes, namely periostin (*Postn*), asporin (*Aspn*), SPARC-like 1 (*Sparcl1*), melanoma cell adhesion molecule (*Mcam*), platelet derived growth factor receptor beta (*Pdgfrb*), fascin homolog 1 (*Fscn1*) and prostate transmembrane protein, androgen induced 1 (*Pmepa1*) were analyzed.

The mRNA expression of all 7 genes was significantly higher in the stroma of C4-2B and VCaP xenografts compared to contralateral bones. When compared to sham-operated bones, the up-regulation of *Aspn* and *Mcam* in C4-2B xenografts and of *Postn* and *Aspn* in VCaP xenografts was not statistically significant. The latter observation is in agreement with the microarray data, which indicated only a trend in *Aspn* up-regulation. There was no difference in mRNA expression of all these genes between contralateral tibiae of the PCa cell-xenografted animals and tibiae of intact animals (not shown), indicating a local rather than a systemic response to bone-xenografted cancer cells.

Furthermore, we confirmed by immunohistochemistry on sections of sham-operated and PCa cell-xenografted bones the stromal expression of Aspn and Postn, 2 proteins encoded by up-regulated genes of the OB-BMST (Supporting File, Figure 1).

Taken together the results from the literature survey and gene expression analysis show the reliability of our approach to analyze specifically the stroma compartment.

**Identification of enriched biological processes**

To identify key biological processes of the OB-BMST, the 321 common probe sets were analyzed for enriched Gene Ontology (GO) terms and functional networks.

Affymetrix probe ids were imported to the Database for Annotation, Visualization and Integrated Discovery (DAVID) (version number 6.7) [4,5]. The functional annotation tool was used to analyze enriched gene ontology terms. In all graphs enriched biological processes with a FDR < 0.5 were visualized.

Protein networks were generated using STRING (version number 9.05, http://string-db.org/). The active prediction methods used were experimental evidence, databases and textmining. Disconnected nodes were hidden in all figures. The confidence score of predicted protein interactions was set to 0.4. The network visualization was done with STRING default options.

The interaction partners highlighted by the STRING analysis were assigned to biological processes according to BioGPS platform (http://biogps.org/).

**Curation strategy to obtain a bone-specific stroma response signature**

The curation strategy is depicted in **Figure 1A.**

OB-BMST gene symbols were used, as most published gene signatures provided gene symbols. Consequently, in case of multiple annotations for one probe set id, gene symbols were kept for all possible annotations. All numbers during the curation of the OB-BMST do not reflect the number of excluded probe set ids. At the end, we merged all remaining gene symbols to the original list, which allows us to state the number of probe set ids kept after curation of the OB-BMST. Furthermore, up- and down-regulated gene symbols were compared separately, to exclude only gene symbols that showed the same direction of regulation. VCaP and C4-2B gene symbols were then converted to human gene symbol orthologs using Biomart (version 0.7). This resulted in the following human gene symbol entries: 435 for VCaP upregulated genes (Supporting File, human orthologs, Table 6a), 153 for VCaP downregulated genes (Supporting File, human orthologs, Table 6b), 325 for C4-2B upregulated genes (Supporting File, human orthologs, table 6c) and 294 for C4-2B downregulated genes (Supporting File, human orthologs, table 6d).

Publicly available gene signatures were either were downloaded from Oncomine (version 4.4.4.3) or directly obtained from NCBI.

***A)*** *The OB-BMST partially overlaps with the desmoplastic and inflammatory response signatures.* Gene signatures used are summarized in Supporting File Table 7. The comparison revealed that 6 genes of the 562 up-regulated human orthologs of the OB-BMST overlap with wound healing (core serum response, CSR) [6], 41 genes with inflammatory [7,8], 32 genes with desmoplastic (desmoid-type fibromatosis, DTF) [9] and 6 genes with CSF1 [10] responses (Supporting file Table 8). *POSTN*, one gene of the OB-BMST “7-gene” list, was also shared by the DTF gene list, indicating that its expression is part of the universal desmoplastic response to tumors [9]. Subtraction of the overlapping genes generated a “Curated 1” OB-BMST list to be further processed.

***B)*** *The “Curated 1” OB-BMST gene list partially overlaps with gene signatures from non-osteotropic cancers.* Further subtraction of stroma signatures derived from cancers that do not or rarely metastasize to bone (Supporting File Table 9), namely gastric [11], pancreatic [12,13], colorectal [14] and esophageal [15,16], revealed an overlap of 61 up-regulated and 18 down-regulated genes of the OB-BMST(Supporting file Table 8). Two further genes from the OB-BMST “7-gene” list, namely *PDGFRB* [12] and *PMEPA1* [11] were common to the stroma signature of non-osteotropic cancers. This observation shows evidence of a cancer-type independent response within the OB-BMST. Subtraction of the full list of overlapping genes generated a further “Curated 2” OB-BMST.

***C)*** *The “Curated 2” OB-BMST gene list partially overlaps with gene signatures from osteotropic cancers.* Several studies have reported the stroma gene expression profile in primary PCa [17-21] (Supporting File Table 10) and MCa [20,22-25] (Supporting File Table 11). A total of 88 differentially regulated genes (70 up- and 18 down-regulated) overlapped between these signatures and the “Curated 2” OB-BMST (grey area in **Figure 2A** and Supporting file Table 8). Three of these 88 genes were also part of the OB-BMST “7-gene” list, namely *ASPN* [20,25] *MCAM* [22] and *SPARCL1* [22,23].

**Comparison with SC niche signatures**

We compared the OB-BMST and the Core OB-BMST with two publicly available signatures, namely the hematopoietic stem cell (HSC) niche signature (HSC-supportive BM stroma cell lines) [26] and the developing prostate SC niche signature (uro-genital mesenchyme, UGM) [27] (**Figure 4 A-D, Table 4, Supporting table S5**).

**References**

1. Schwaninger R, Rentsch CA, Wetterwald A, van der Horst G, van Bezooijen RL, et al. (2007) Lack of noggin expression by cancer cells is a determinant of the osteoblast response in bone metastases. Am J Pathol 170: 160–175. doi:10.2353/ajpath.2007.051276.

2. Irizarry RA, Bolstad BM, Collin F, Cope LM, Hobbs B, et al. (2003) Summaries of Affymetrix GeneChip probe level data. Nucleic Acids Research 31: e15.

3. Gallicano GI, Bauer C, Fuchs E (2001) Rescuing desmoplakin function in extra-embryonic ectoderm reveals the importance of this protein in embryonic heart, neuroepithelium, skin and vasculature. Development 128: 929–941.

4. Huang DW, Sherman BT, Lempicki RA (2009) Bioinformatics enrichment tools: paths toward the comprehensive functional analysis of large gene lists. Nucleic Acids Research 37: 1–13. doi:10.1093/nar/gkn923.

5. Huang DW, Sherman BT, Lempicki RA (2009) Systematic and integrative analysis of large gene lists using DAVID bioinformatics resources. Nat Protoc 4: 44–57. doi:10.1038/nprot.2008.211.

6. Chang HY, Sneddon JB, Alizadeh AA, Sood R, West RB, et al. (2004) Gene expression signature of fibroblast serum response predicts human cancer progression: similarities between tumors and wounds. PLoS Biol 2: E7. Available: http://eutils.ncbi.nlm.nih.gov/entrez/eutils/elink.fcgi?dbfrom=pubmed&id=14737219&retmode=ref&cmd=prlinks.

7. Cooper CR, Chay CH, Gendernalik JD, Lee H-L, Bhatia J, et al. (2003) Stromal factors involved in prostate carcinoma metastasis to bone. Cancer 97: 739–747. Available: http://doi.wiley.com/10.1002/cncr.11181.

8. Stein T, Morris JS, Davies CR, Weber-Hall SJ, Duffy M-A, et al. (2004) Involution of the mouse mammary gland is associated with an immune cascade and an acute-phase response, involving LBP, CD14 and STAT3. Breast Cancer Res 6: R75–R91. doi:10.1186/bcr753.

9. Beck AH, Espinosa I, Gilks CB, van de Rijn M, West RB (2008) The fibromatosis signature defines a robust stromal response in breast carcinoma. Lab Invest 88: 591–601. doi:10.1038/labinvest.2008.31.

10. Beck AH, Espinosa I, Edris B, Li R, Montgomery K, et al. (2009) The macrophage colony-stimulating factor 1 response signature in breast carcinoma. Clin Cancer Res 15: 778–787. doi:10.1158/1078-0432.CCR-08-1283.

11. Cho JY, Lim JY, Cheong JH, Park Y-Y, Yoon S-L, et al. (2011) Gene expression signature-based prognostic risk score in gastric cancer. Clin Cancer Res 17: 1850–1857. doi:10.1158/1078-0432.CCR-10-2180.

12. Binkley CE, Zhang L, Greenson JK, Giordano TJ, Kuick R, et al. (2004) The molecular basis of pancreatic fibrosis: common stromal gene expression in chronic pancreatitis and pancreatic adenocarcinoma. Pancreas 29: 254–263.

13. Buchholz M, Braun M, Heidenblut A, Kestler HA, Klöppel G, et al. (2005) Transcriptome analysis of microdissected pancreatic intraepithelial neoplastic lesions. Oncogene 24: 6626–6636. doi:10.1038/sj.onc.1208804.

14. Sabates-Bellver J, Van der Flier LG, de Palo M, Cattaneo E, Maake C, et al. (2007) Transcriptome profile of human colorectal adenomas. Mol Cancer Res 5: 1263–1275. doi:10.1158/1541-7786.MCR-07-0267.

15. Saadi A, Shannon NB, Lao-Sirieix P, O'Donovan M, Walker E, et al. (2010) Stromal genes discriminate preinvasive from invasive disease, predict outcome, and highlight inflammatory pathways in digestive cancers. Proc Natl Acad Sci USA 107: 2177–2182. doi:10.1073/pnas.0909797107.

16. Hao Y, Triadafilopoulos G, Sahbaie P, Young HS, Omary MB, et al. (2006) Gene expression profiling reveals stromal genes expressed in common between Barrett's esophagus and adenocarcinoma. YGAST 131: 925–933. doi:10.1053/j.gastro.2006.04.026.

17. Bacac M, Provero P, Mayran N, Stehle J-C, Fusco C, et al. (2006) A Mouse Stromal Response to Tumor Invasion Predicts Prostate and Breast Cancer Patient Survival. PLoS ONE 1: e32. doi:10.1371/journal.pone.0000032.s013.

18. Dakhova O, Ozen M, Creighton CJ, Li R, Ayala G, et al. (2009) Global Gene Expression Analysis of Reactive Stroma in Prostate Cancer. Clinical Cancer Research 15: 3979–3989. Available: http://clincancerres.aacrjournals.org/cgi/doi/10.1158/1078-0432.CCR-08-1899.

19. Lapointe J, Li C, Higgins JP, van de Rijn M, Bair E, et al. (2004) Gene expression profiling identifies clinically relevant subtypes of prostate cancer. Proc Natl Acad Sci U S A 101: 811–816. doi:10.1073/pnas.0304146101.

20. Planche A, Bacac M, Provero P, Fusco C, Delorenzi M, et al. (2011) Identification of prognostic molecular features in the reactive stroma of human breast and prostate cancer. PLoS ONE 6: e18640. doi:10.1371/journal.pone.0018640.

21. Richardson AM, Woodson K, Wang Y, Rodriguez-Canales J, Erickson HS, et al. (2007) Global expression analysis of prostate cancer-associated stroma and epithelia. Diagn Mol Pathol 16: 189–197. doi:10.1097/PDM.0b013e3180de20ac.

22. Allinen M, Beroukhim R, Cai L, Brennan C, Lahti-Domenici J, et al. (2004) Molecular characterization of the tumor microenvironment in breast cancer. Cancer Cell 6: 17–32. doi:10.1016/j.ccr.2004.06.010.

23. Finak G, Bertos N, Pepin F, Sadekova S, Souleimanova M, et al. (2008) Stromal gene expression predicts clinical outcome in breast cancer. Nat Med 14: 518–527. doi:10.1038/nm1764.

24. Karnoub AE, Dash AB, Vo AP, Sullivan A, Brooks MW, et al. (2007) Mesenchymal stem cells within tumour stroma promote breast cancer metastasis. Nature 449: 557–563. doi:10.1038/nature06188.

25. Ma X-J, Dahiya S, Richardson E, Erlander M, Sgroi DC (2009) Gene expression profiling of the tumor microenvironment during breast cancer progression. Breast Cancer Res 11: R7. doi:10.1186/bcr2222.

26. Charbord P, Moore KA (2005) Gene Expression in Stem Cell-Supporting Stromal Cell Lines. Ann N Y Acad Sci 1044: 159–167. doi:10.1196/annals.1349.020.

27. Blum R, Gupta R, Burger PE, Ontiveros CS, Salm SN, et al. (2010) Molecular Signatures of the Primitive Prostate Stem Cell Niche Reveal Novel Mesenchymal-Epithelial Signaling Pathways. PLoS ONE 5: e13024. doi:10.1371/journal.pone.0013024.s005.

**Legends to Figure and Tables of the Supporting File**

**Figure 1. Periostin and Asporin are expressed in the stroma of osteoinductive cancer cells xenografts.** In sham-operated tibiae (**A**), Postn immunoreactivity was found exclusively in periosteal OBs and putative OB precursors. In PCa cell-xenografted bones (**C**), a strong Postn immunoreactivity covered a larger longitudinal surface of the periosteum and was also extended to cells of the fibrous layer. Cancer cells were negative.

In sham-operated bones (**B**), Aspn immunoreactivity was found in chondrocytes, in periosteal OB precursors and in few OBs of the primary spongiosa. Occasional Aspn-positive pericyte-like cells were observed around capillaries within the bone healing reaction to the surgical drilling (not shown). In PCa cell-xenografted bones (**D**), Aspn immunoreactivity was evident in the osteogenic layer of the entire periosteum of the bone cortex facing the intramedullary tumor growth and in the tumor stroma. Intense Aspn immunoreactivity was observed in pericyte-like cells surrounding tumor stroma capillaries in areas of strong OB-response. Mature OBs and cancer cells were invariably negative. Insets represent a higher magnification of selected areas. Scale bar = 100 *μ*m. Abbreviations: Postn, periostin; Aspn, asporin.

**Table 1.** List of excluded cross-hybridising probe sets and corresponding genes.

**Table 2**. Gene expression assays used in this study.

**Table 3.** Primary antibodies used for immunohistochemistry.

**Table 4.** List of pan-epithelial/prostate epithelial cell-specific marker genes used to assess the reliability of TCTP in dissecting the stroma-specific transcriptome in PCa xenograft models.

**Table 5**. Literature review of selected stromal genes.

**Table 6.** List of human orthologs of the OB-BMST.

**Table 7.** Lists of stroma gene signatures from inflammatory/wound healing and desmoplastic responses.

**Table 8.** List of overlapping OB-BMST genes with inflammatory/wound healing and desmoplastic response signatures, and from non-osteotropic and osteotropic cancers.

**Table 9.** List of non-osteotropic cancer stroma signatures.

**Table 10.** List of PCa stroma gene signatures.

**Table 11.** List of MCa stroma gene signatures.
