# Supplementary material for: The Molecular Signature of the Stroma Response in Prostate Cancer-Induced Osteoblastic Bone Metastasis Highlights Expansion of Hematopoietic and Prostate Epithelial Stem Cell Niches
Source: PLoS One. 2014 Dec 8;9(12):e114530. doi: 10.1371/journal.pone.0114530 (PMC4259356; doi:10.1371/journal.pone.0114530)
Supplement: S1 File — This file contains an extended version of the Material and Methods, including references, one Figure and 11 Tables. (ZIP) [file pone.0114530.s010.zip › Supporting File/File S1. Table 2.doc]

**File S1. Table 2A.** Mouse gene expression assays used in this study.

| Gene | Assay ^a)^ | Human tissue/cells used to  assess species specificity |
| --- | --- | --- |
| *Actb* | Mm02619580_g1 |  |
| *Aspn* | Mm00445945_m1 | Cartilage |
| *B2m* | Mm00437762_m1 |  |
| *Bst1* | Mm00477672_m1 | Whole blood |
| *Cd109* | Mm00462151_m1 |  |
| *Dedd2* | Mm01149726_m1 | VCaP prostate cancer cells |
| *Epha3* | Mm00580743_m1 |  |
| *Fscn1* | Mm00456046_m1 |  |
| *Hprt1* | Mm00446968_m1 |  |
| *Mcam* | Mm00522397_m1 | PC-3 prostate cancer cells |
| *Pdgfrb* | Mm01262489_m1 | Aorta |
| *Pmepa1* | Mm00452229_m1 | C4-2B4 prostate cancer cells |
| *Postn* | Mm00450111_m1 | MG63 osteoblasts |
| *Ptn* | Mm01132688_m1 |  |
| *Slit3* | Mm01326992_m1 |  |
| *Sparcl1* | Mm00447780_m1 | Colon |
| NOTE: ^a)^ TaqMan gene expression assay, Applied Biosystems | | |

**Supporting File. Table 2B.** Human gene expression assays used in this study.

| Gene | Assay ^a)^ |  |
| --- | --- | --- |
| 18S | Hs99999901_s1 |  |
| *ACTB* | Hs99999903_m1 |  |
| *ASPN* | Hs00214395_m1 |  |
| *B2M* | Hs99999907_m1 |  |
| *BST1* | Hs01070190_m1 |  |
| *DEDD2* | Hs00958059_m1 |  |
| *FSCN1* | Hs 00602051_m1 |  |
| *HPRT1* | Hs99999909_m1 |  |
| *MCAM* | Hs00174838_m1 |  |
| *PDGFRB* | Hs00182163_m1 |  |
| *PMEPA1* | Hs03987603_g1 |  |
| *POSTN* | Hs00170815_m1 |  |
| *SPARCL1* | Hs00190740_m1 |  |
| NOTE: ^a)^ TaqMan gene expression assay, Applied Biosystems | | |
