# Supplementary material for: The Molecular Signature of the Stroma Response in Prostate Cancer-Induced Osteoblastic Bone Metastasis Highlights Expansion of Hematopoietic and Prostate Epithelial Stem Cell Niches
Source: PLoS One. 2014 Dec 8;9(12):e114530. doi: 10.1371/journal.pone.0114530 (PMC4259356; doi:10.1371/journal.pone.0114530)
Supplement: S1 File — This file contains an extended version of the Material and Methods, including references, one Figure and 11 Tables. (ZIP) [file pone.0114530.s010.zip › Supporting File/File S1. Table 3.doc]

**File S1. Table 3**. Primary antibodies used for immunohistochemistry.

| Antibodies | Company | Clone | Concentration^a^ | Antigen retrieval | |
| --- | --- | --- | --- | --- | --- |
| Postn | R&D | 355613 | 10 μg/ml | None | |
| POSTN | Abcam |  | 0.25 μg/ml | EDTA | |
| Aspn/ASPN | Abcam |  | 10 μg/ml | None | |
| FSCN1 | Abcam |  | 10 μg/ml | None | |
| PTN | Santa Cruz |  | 5 μg/ml | Citrate | |
| EPHA3 | Santa Cruz |  | 0.2 μg/ml | Citrate | |
| ACTA2 | Sigma-Aldrich | 1A4 | 1:2000 | None | |
| CHGA | DAKO | A0430 | 5 μg/ml | None | |
| SYP | Invitrogen | Z66 | 5 μg/ml | None | |
| Note: ^a)^ Working concentrations are indicated where known; dilutions are given where antibody concentration is not specified by the manufacturer.  Abbreviations: Postn/POSTN, periostin; Aspn/ASPN, asporin; FSCN1, fascin homolog 1; PTN, pleiotrophin; EPHA3, Eph receptor A3; ACTA2, actin α2 smooth muscle, aorta; CHGA, chromogranin A; SYP, synaptophysin. | | | | |  |
