# Supplementary material for: The Molecular Signature of the Stroma Response in Prostate Cancer-Induced Osteoblastic Bone Metastasis Highlights Expansion of Hematopoietic and Prostate Epithelial Stem Cell Niches
Source: PLoS One. 2014 Dec 8;9(12):e114530. doi: 10.1371/journal.pone.0114530 (PMC4259356; doi:10.1371/journal.pone.0114530)
Supplement: S1 File — This file contains an extended version of the Material and Methods, including references, one Figure and 11 Tables. (ZIP) [file pone.0114530.s010.zip › Supporting File/File S1. Table 4 .doc]

**File S1. Table 4.** List of pan-epithelial/prostate epithelial cell-specific marker genes used to assess the reliability of TCTP in dissecting the stroma-specific transcriptome in PCa xenograft models.

| **Gene symbol** | **Gene name** |
| --- | --- |
| **Afp** | Alpha fetoprotein |
| **Ar** | Androgen receptor |
| **Cdh1** | Cadherin 1 |
| **Cldn1** | Claudin 1 |
| **Cldn3** | Claudin 3 |
| **Cldn4** | Claudin 4 |
| **Cldn5** | Claudin 5 |
| **Cldn7** | Claudin 7 |
| **Cldn8** | Claudin 8 |
| **Cldn10** | Claudin 10 |
| **Eno2** | Enolase 2, gamma, neuronal |
| **Epcam** | Epithelial cell adhesion molecule |
| **Jup** | Junction plakoglobin |
| **Kit** | Kit oncogene |
| **Klkb1** | Kallikrein B, plasma 1 |
| **Krt5** | Keratin 5 |
| **Krt8** | Keratin 8 |
| **Krt14** | Keratin 14 |
| **Krt18** | Keratin 18 |
| **Krt19** | Keratin 19 |
| **Nkx3-1** | NK-3 transcription factor, locus 1 |
| **Prom1 (Cd133)** | Prominin 1 |
| **Psca** | Prostate stem cell antigen |
| **Steap1** | Six transmembrane epithelial antigen of the prostate 1 |
| **Syp** | Synaptophysin |
| **Tjp1** | Tight junction protein 1 |

Gene names according to Mouse Genome Informatics: <http://www.informatics.jax.org/>
