# Supplementary material for: The Molecular Signature of the Stroma Response in Prostate Cancer-Induced Osteoblastic Bone Metastasis Highlights Expansion of Hematopoietic and Prostate Epithelial Stem Cell Niches
Source: PLoS One. 2014 Dec 8;9(12):e114530. doi: 10.1371/journal.pone.0114530 (PMC4259356; doi:10.1371/journal.pone.0114530)
Supplement: S1 File — This file contains an extended version of the Material and Methods, including references, one Figure and 11 Tables. (ZIP) [file pone.0114530.s010.zip › Supporting File/File S1. Table 5.doc]

**File S1. Table 5.** Literature review of selected stromal genes.

| **Gene** | **Protein name** | **Confirmed expression in stroma** |
| --- | --- | --- |
| **Aspn** | Asporin | Orr B *et al.* 2011[1]  Ma XJ *et al.* 2009[2] |
| **Postn** | Periostin | Malanchi I *et al.* 2012[3]  Planche A *et al.* 2011[4]  Kii I *et al.* 2010[5]  Horiuchi K *et al.* 1999[6] |
| **Sparcl1** | Sparc-like 1 | Turtoi A *et al.* 2012[7]  Framson PE, Sage EH. 2004[8]  Sullivan MM *at al.* 2008[9] |
| **Fscn1** | Fascin homolog 1 | Nese N *et al.* 2010[10]  Hashimoto Y *et al.* 2011[11]  Hashimoto Y *et al.* 2008[12] |
| **Pdgfrb** | Platelet derived growth factor  receptor, beta polypeptide | Ostman A and Augsten M 2009[13]  Yuzawa S *et al.* 2012[14]  Catena R *et al.* 2011[15] |
| **Mcam** | Melanoma cell adhesion molecule | Sacchetti B *et al.* 2007[16]  Despars G *et al.* 2008[17]  Trevino-Villarreal JH *at al.* 2011[18] |
| **Fmod** | Fibromodulin | Ichii M *et al.* 2012[19]  Coulson-Thomas VJ *et al.* 2010[20]  Oldberg A *et al.* 2007[21] |
| **Serpinh1** | Serine peptidase inhibitor 1 | Masago Y *et al.* 2012[22]  Alt E *et al.* 2011[23]  Kuroda K, Tajima S. 2008[24] |
| **Thbs2** | Thrombospondin 2 | Jeong SY *et al.* 2013[25]  Matsuba M *et al.* 2011[26]  Zaslavsky A *et al.* 2010[27] |
| **Timp1** | Tissue inhibitor of metalloproteinase 1 | Zhang Q *et al.* 2012[28]  Knowles JP *et al.* 2012[29]  Untergasser G *et al.* 2005[30] |
| **Epha3** | Eph receptor A3 | Ting MJ *et al.* 2010[31] |
| **Prss23** | Protease, serine, 23 | Wahlberg P *et al.* 2008[32] |
| **Mmp14** | Matrix metallopeptidase 14 | Ma XJ *et al.* 2009[2]  Coulson-Thomas VJ *et al.* 2010[20] |
| **Tgfb3** | Transforming growth factor, beta 3 | West RB *et al.* 2005[33]  Blum R *et al.* 2010[34]  Farmer P *et al.* 2009[35] |
| **Cd276** | CD276 antigen | Al-Nbaheem M *et al.* 2012[36]  Xu L *et al.* 2011[37]  Sun J *et al.* 2010[38] |

Gene and protein names according to Mouse Genome Informatics: <http://www.informatics.jax.org/>

**References:**

1.	Orr B, Riddick ACP, Stewart GD, Anderson RA, Franco OE, et al. (2011) Identification of stromally expressed molecules in the prostate by tag-profiling of cancer-associated fibroblasts, normal fibroblasts and fetal prostate. Oncogene 31: 1130–1142. doi:10.1038/onc.2011.312.

2.	Ma X-J, Dahiya S, Richardson E, Erlander M, Sgroi DC (2009) Gene expression profiling of the tumor microenvironment during breast cancer progression. Breast Cancer Res 11: R7. doi:10.1186/bcr2222.

3.	Malanchi I, Santamaria-Martínez A, Susanto E, Peng H, Lehr H-A, et al. (2012) Interactions between cancer stem cells and their niche govern metastatic colonization. Nature 481: 85–89. Available: http://www.nature.com/doifinder/10.1038/nature10694.

4.	Planche A, Bacac M, Provero P, Fusco C, Delorenzi M, et al. (2011) Identification of prognostic molecular features in the reactive stroma of human breast and prostate cancer. PLoS ONE 6: e18640. doi:10.1371/journal.pone.0018640.

5.	Kii I, Nishiyama T, Li M, Matsumoto K-I, Saito M, et al. (2010) Incorporation of tenascin-C into the extracellular matrix by periostin underlies an extracellular meshwork architecture. J Biol Chem 285: 2028–2039. Available: http://www.jbc.org/cgi/doi/10.1074/jbc.M109.051961.

6.	Horiuchi K, Amizuka N, Takeshita S, Takamatsu H, Katsuura M, et al. (1999) Identification and characterization of a novel protein, periostin, with restricted expression to periosteum and periodontal ligament and increased expression by transforming growth factor beta. J Bone Miner Res 14: 1239–1249. doi:10.1359/jbmr.1999.14.7.1239.

7.	Turtoi A, Musmeci D, Naccarato AG, Scatena C, Ortenzi V, et al. (2012) Sparc-Like Protein 1 Is a New Marker of Human Glioma Progression. J Proteome Res 11: 5011–5021. doi:10.1021/pr3005698.

8.	Framson PE, Sage EH (2004) SPARC and tumor growth: where the seed meets the soil? J Cell Biochem 92: 679–690. doi:10.1002/jcb.20091.

9.	Sullivan MM, Puolakkainen PA, Barker TH, Funk SE, Sage EH (2008) Altered tissue repair in hevin-null mice: inhibition of fibroblast migration by a matricellular SPARC homolog. Wound repair and regeneration : official publication of the Wound Healing Society [and] the European Tissue Repair Society 16: 310–319. doi:10.1111/j.1524-475X.2008.00370.x.

10.	Nese N, Kandiloglu AR, Simsek G, Lekili M, Ozdamar A, et al. (2010) Comparison of the desmoplastic reaction and invading ability in invasive ductal carcinoma of the breast and prostatic adenocarcinoma based on the expression of heat shock protein 47 and fascin. Anal Quant Cytol Histol 32: 90–101.

11.	Hashimoto Y, Kim DJ, Adams JC (2011) The roles of fascins in health and disease. J Pathol 224: 289–300. doi:10.1002/path.2894.

12.	Hashimoto Y, Skacel M, Adams JC (2008) Association of loss of epithelial syndecan-1 with stage and local metastasis of colorectal adenocarcinomas: an immunohistochemical study of clinically annotated tumors. BMC Cancer 8: 185. doi:10.1186/1471-2407-8-185.

13.	Ostman A, Augsten M (2009) Cancer-associated fibroblasts and tumor growth--bystanders turning into key players. Curr Opin Genet Dev 19: 67–73. doi:10.1016/j.gde.2009.01.003.

14.	Yuzawa S, Kano MR, Einama T, Nishihara H (2012) PDGFRβ expression in tumor stroma of pancreatic adenocarcinoma as a reliable prognostic marker. Med Oncol 29: 2824–2830. doi:10.1007/s12032-012-0193-0.

15.	Catena R, Luis-Ravelo D, Antón I, Zandueta C, Salazar-Colocho P, et al. (2011) PDGFR signaling blockade in marrow stroma impairs lung cancer bone metastasis. Cancer Res 71: 164–174. doi:10.1158/0008-5472.CAN-10-1708.

16.	Sacchetti B, Funari A, Michienzi S, Di Cesare S, Piersanti S, et al. (2007) Self-renewing osteoprogenitors in bone marrow sinusoids can organize a hematopoietic microenvironment. Cell 131: 324–336. Available: http://linkinghub.elsevier.com/retrieve/pii/S0092867407010872.

17.	Despars G, Periasamy P, Tan J, Abbey J, O'Neill TJ, et al. (2008) Gene signature of stromal cells which support dendritic cell development. Stem cells and development 17: 917–927. doi:10.1089/scd.2007.0170.

18.	Treviño-Villarreal JH, Cotanche DA, Sepúlveda R, Bortoni ME, Manneberg O, et al. (2011) Host-derived pericytes and Sca-1+ cells predominate in the MART-1- stroma fraction of experimentally induced melanoma. Journal of Histochemistry & Cytochemistry 59: 1060–1075. doi:10.1369/0022155411428078.

19.	Ichii M, Frank MB, Iozzo RV, Kincade PW (2012) The canonical Wnt pathway shapes niches supportive of hematopoietic stem/progenitor cells. Blood 119: 1683–1692. doi:10.1182/blood-2011-07-369199.

20.	Coulson-Thomas VJ, Gesteira TF, Coulson-Thomas YM, Vicente CM, Tersariol ILS, et al. (2010) Fibroblast and prostate tumor cell cross-talk: fibroblast differentiation, TGF-β, and extracellular matrix down-regulation. Exp Cell Res 316: 3207–3226. doi:10.1016/j.yexcr.2010.08.005.

21.	Oldberg A, Kalamajski S, Salnikov AV, Stuhr L, Mörgelin M, et al. (2007) Collagen-binding proteoglycan fibromodulin can determine stroma matrix structure and fluid balance in experimental carcinoma. Proc Natl Acad Sci U S A 104: 13966–13971. doi:10.1073/pnas.0702014104.

22.	Masago Y, Hosoya A, Kawasaki K, Kawano S, Nasu A, et al. (2012) The molecular chaperone Hsp47 is essential for cartilage and endochondral bone formation. J Cell Sci 125: 1118–1128. doi:10.1242/jcs.089748.

23.	Alt E, Yan Y, Gehmert S, Song Y-H, Altman A, et al. (2011) Fibroblasts share mesenchymal phenotypes with stem cells, but lack their differentiation and colony-forming potential. Biol Cell 103: 197–208. doi:10.1042/BC20100117.

24.	Kuroda K, Tajima S (2008) Proliferation of HSP47-positive skin fibroblasts in dermatofibroma. J Cutan Pathol 35: 21–26. doi:10.1111/j.1600-0560.2007.00768.x.

25.	Jeong SY, Kim DH, Ha J, Jin HJ, Kwon S-J, et al. (2013) Thrombospondin-2 Secreted by Human Umbilical Cord Blood-Derived Mesenchymal Stem Cells Promotes Chondrogenic Differentiation. Stem Cells. doi:10.1002/stem.1471.

26.	Matsuba M, Hutcheon AEK, Zieske JD (2011) Localization of thrombospondin-1 and myofibroblasts during corneal wound repair. Exp Eye Res 93: 534–540. doi:10.1016/j.exer.2011.06.018.

27.	Zaslavsky A, Chen C, Grillo J, Baek K-H, Holmgren L, et al. (2010) Regional control of tumor growth. Molecular Cancer Research 8: 1198–1206. doi:10.1158/1541-7786.MCR-10-0047.

28.	Zhang Q, Liu S, Ge D, Zhang Q, Xue Y, et al. (2012) Interleukin-17 promotes formation and growth of prostate adenocarcinoma in mouse models. Cancer Res 72: 2589–2599. doi:10.1158/0008-5472.CAN-11-3795.

29.	Knowles JP, Shi-Wen X, Haque S-U, Bhalla A, Dashwood MR, et al. (2012) Endothelin-1 stimulates colon cancer adjacent fibroblasts. Int J Cancer 130: 1264–1272. doi:10.1002/ijc.26090.

30.	Untergasser G, Gander R, Lilg C, Lepperdinger G, Plas E, et al. (2005) Profiling molecular targets of TGF-beta1 in prostate fibroblast-to-myofibroblast transdifferentiation. Mech Ageing Dev 126: 59–69. doi:10.1016/j.mad.2004.09.023.

31.	Ting MJ, Day BW, Spanevello MD, Boyd AW (2010) Activation of ephrin A proteins influences hematopoietic stem cell adhesion and trafficking patterns. Exp Hematol 38: 1087–1098. doi:10.1016/j.exphem.2010.07.007.

32.	Wahlberg P, Nylander A, Ahlskog N, Liu K, Ny T (2008) Expression and localization of the serine proteases high-temperature requirement factor A1, serine protease 23, and serine protease 35 in the mouse ovary. Endocrinology 149: 5070–5077. doi:10.1210/en.2007-1736.

33.	West RB, Nuyten DSA, Subramanian S, Nielsen TO, Corless CL, et al. (2005) Determination of Stromal Signatures in Breast Carcinoma. PLoS Biol 3: e187. doi:10.1371/journal.pbio.0030187.st004.

34.	Blum R, Gupta R, Burger PE, Ontiveros CS, Salm SN, et al. (2010) Molecular Signatures of the Primitive Prostate Stem Cell Niche Reveal Novel Mesenchymal-Epithelial Signaling Pathways. PLoS ONE 5: e13024. doi:10.1371/journal.pone.0013024.s005.

35.	Farmer P, Bonnefoi H, Anderle P, Cameron D, Wirapati P, et al. (2009) A stroma-related gene signature predicts resistance to neoadjuvant chemotherapy in breast cancer. Nat Med 15: 68–74. doi:10.1038/nm.1908.

36.	Al-Nbaheen M, Vishnubalaji R, Ali D, Bouslimi A, Al-Jassir F, et al. (2012) Human stromal (mesenchymal) stem cells from bone marrow, adipose tissue and skin exhibit differences in molecular phenotype and differentiation potential. Stem Cell Rev 9: 32–43. doi:10.1007/s12015-012-9365-8.

37.	Xu L, Zhang G, Zhou Y, Chen Y, Xu W, et al. (2011) Stimulation of B7-H3 (CD276) directs the differentiation of human marrow stromal cells to osteoblasts. Immunobiology 216: 1311–1317. doi:10.1016/j.imbio.2011.05.013.

38.	Sun J, Chen L-J, Zhang G-B, Jiang J-T, Zhu M, et al. (2010) Clinical significance and regulation of the costimulatory molecule B7-H3 in human colorectal carcinoma. Cancer Immunol Immunother 59: 1163–1171. doi:10.1007/s00262-010-0841-1.
